# Description and ontogeny of a 40-million-year-old parasitic isopodan crustacean: *Parvucymoides dvorakorum* gen. et sp. nov.

**DOI:** 10.7717/peerj.12317

**Published:** 2021-12-09

**Authors:** Serita Van der Wal, Mario Schädel, Boris Ekrt, Joachim T. Haug

**Affiliations:** 1 Zoomorphology Group, Faculty of Biology, LMU Munich, Planegg-Martinsried, Germany; 2 Department of Paleontology, National Museum, Prague, Czech Republic; 3 GeoBio-Center, LMU Munich, Munich, Germany

**Keywords:** Cymothoida, fossil Cymothoidae, fish parasite, Eocene, Kučlín

## Abstract

A collection of exceptionally well-preserved fossil specimens of crustaceans, clearly representatives of Isopoda, is presented here. Excavated from the late Eocene (approximately 40 million years ago) freshwater sediments of the Trupelník hill field site near Kučlín, Czech Republic, these specimens are preserved with many details of the appendages. The morphological characteristics of the fossils were documented using macro-photography with polarised light, as well as stereo imaging. These characteristics, especially including the trunk appendage morphology, were compared to those of related extant groups from different ontogenetic stages. All specimens are conspecific, representing a single species *Parvucymoides dvorakorum* gen. et sp. nov. Morphometric analysis of body shapes and sizes of the reconstructed fossils and related extant species were performed. These analyses provided insight into the ontogenetic stages of each reconstructed fossil specimen. In combination with the morphological assessment, the results indicate that the fossils represent at least two (possibly three) developmental stages, including immatures. The morphology of the appendages suggests that these fossils were parasites. The fossils are interpreted as either representatives of Cymothoidae or at least closely related to this group.

## Introduction

Isopoda is an extremely species-rich and diverse group of organisms (Wilson, 2009; Poore & Bruce, 2012). Among the marine forms of Isopoda, Cymothoida Wägele, 1989 is a morphologically and distributionally diverse group, with a variety of life strategies, ranging from scavengers and predators (see Holdich, 1981; Wilson, Sims & Grutter, 2011; Robin et al., 2019; Youssef et al., 2020) to highly specialised temporary and permanent parasitic individuals (see Hadfield, Smit & Avenant-Oldewage, 2009; Williams & Boyko, 2012; Alves-Júnior et al., 2019). Despite the large number of species and the morphological diversity within extant representatives of Cymothoida (Boyko et al., 2019), the current fossil record does not reflect this diversity (Hyžný, Bruce & Schlögl, 2013; Smit, Bruce & Hadfield, 2014). In most cases, only the dorsal sclerites (tergites) of the posterior body region are preserved as fossils, likely as a result of the biphasic moulting process that characterises Isopoda (Wieder & Feldmann, 1992; Feldmann & Goolaerts, 2005; Hansen & Hansen, 2010; Hyžný, Bruce & Schlögl, 2013; Etter, 2014). Fossil remains of Isopoda are also mostly preserved without complete or accessible appendages, impeding their further systematic interpretation and comparison to extant groups (Hyžný, Bruce & Schlögl, 2013; Smit, Bruce & Hadfield, 2014; Maguire et al., 2018).

The majority of fossil specimens that can be interpreted as representatives of Cymothoida seem to be predatory or scavenging forms. Several of the ingroups of Cymothoida have species that exhibit parasitic strategies (temporarily or permanently) during some stage of development, or for a specific duration of time. Species with parasitic life strategies are found in the following groups: Corallanidae Hansen, 1890 (see Gentil-Vasconcelos & Tavares-Dias, 2015; Nagasawa, Imai & Saito, 2018), Aegidae White, 1850 (see Nair & Nair, 1983; Cavalcanti et al., 2012), Cymothoidae Leach, 1818 (see Kottarathil et al., 2019; Mahmoud, Fahmy & Abuowarda, 2020), Epicaridea (including Bopyroidea Rafinesque, 1815 and Cryptoniscoidea Kossmann, 1880; see Roccatagliata & Jordá, 2002; Alves-Júnior et al., 2019), Gnathiidae Leach, 1814 (see Smit, Basson & Van As, 2003; Marino et al., 2004) and possibly *Urda* Münster, 1840 (see Nagler, Hyžný & Haug, 2017).

Direct indications of parasitic behaviour by representatives of Isopoda (*e.g.*, body fossils of parasites on the suspected host) are scarce. Nagler et al. (2016) described and presented a direct parasite-host interaction from 150 million years old fossils, containing both the host and the interpreted parasitic representatives of Cymothoida attached to it. Less direct indications of parasitic behaviour for Cymothoida include:

(1) Deformations of the host, such as swellings on the shields of fossil crustaceans, can serve as an indication for parasitic behaviour of representatives of Bopyridae (ingroup of Cymothoida; Morris, 1981; Boyko, Williams & Markham, 2012). Records and photographs of these deformations have been provided in, for example, Bachmayer (1948), Radwański (1972), Klompmaker et al. (2014), Klompmaker et al. (2018) and Robins & Klompmaker (2019).

(2) The reconstructed functional morphology of the fossil remains as an indication for possible parasitic behaviour (Nagler & Haug, 2016; Nagler et al., 2016). If the quality of preservation is sufficient, the functional morphology can be reconstructed for isolated fossil remains of representatives of Cymothoida. Here, the attaching appendages, such as the anterior trunk appendages (thoracopods) and mouthparts, are particularly informative.

(3) A specific and distinct life stage, such as a dispersal stage, if it is only known in parasitic species of the modern fauna, is also an indication for parasitic behaviour. For example, the distinct, dispersal larval stages of Epicaridea (epicaridium, microniscium and cryptoniscium), which are unique to the group. Serrano-Sánchez et al. (2016) reported the first direct body fossils (without the host) of cryptoniscium larvae from Miocene Chiapas Amber, originating from Mexico. Shortly thereafter, Néraudeau et al. (2017) and Schädel, Perrichot & Haug (2019) reported on separate additional specimens of epicaridean larvae from Cretaceous French Vendean amber. The latest report of such an indication of parasitic behaviour is provided in Schädel et al. (2021).

(4) A phylogenetic position of which all representatives exhibit a parasitic behaviour is another indication for parasitic behaviour, provided that the supporting morphological characters for parasitism are also accessible. Some previous publications have reported on fossil finds of specimens that might be closely related to Cymothoidae (Bowman, 1971; Nagler et al., 2016), or that could be early forms of Cymothoidae.

Some fossils have been described as species of or closely related to Aegidae, based on similarities with extant species (*e.g.*, Van Straelen, 1930; Hessler, 1969; Polz, 2005; Hansen & Hansen, 2010). *Urda*, a group of species associated with fossil fish, has recently been interpreted as an ingroup of Cymothoida, based on the functional morphology of its representatives (Nagler, Hyžný & Haug, 2017).

Here we present exceptionally well-preserved fossil representatives and describe a new species of Cymothoida that provide clear indications for parasitic behaviour, based on morphology and systematic interpretation. We compare the morphological characters, body shapes and sizes of these fossils, with those of extant genera and species. These comparisons provide some insight into the possible behaviour and ontogenetic variability of the fossils.

## Materials & Methods

### Material

The examined fossil specimens were collected from Kučlín, Czech Republic (Fig. 1), during 1995–2010 by Zdeněk Dvořák and Pavel Dvořák. A total of 11 fossil specimens were examined, photographed and illustrated in detail (Figs. 2–18). All specimens are deposited at the National Museum, Prague, under collection numbers P2338–P2348.

**Figure 1:**
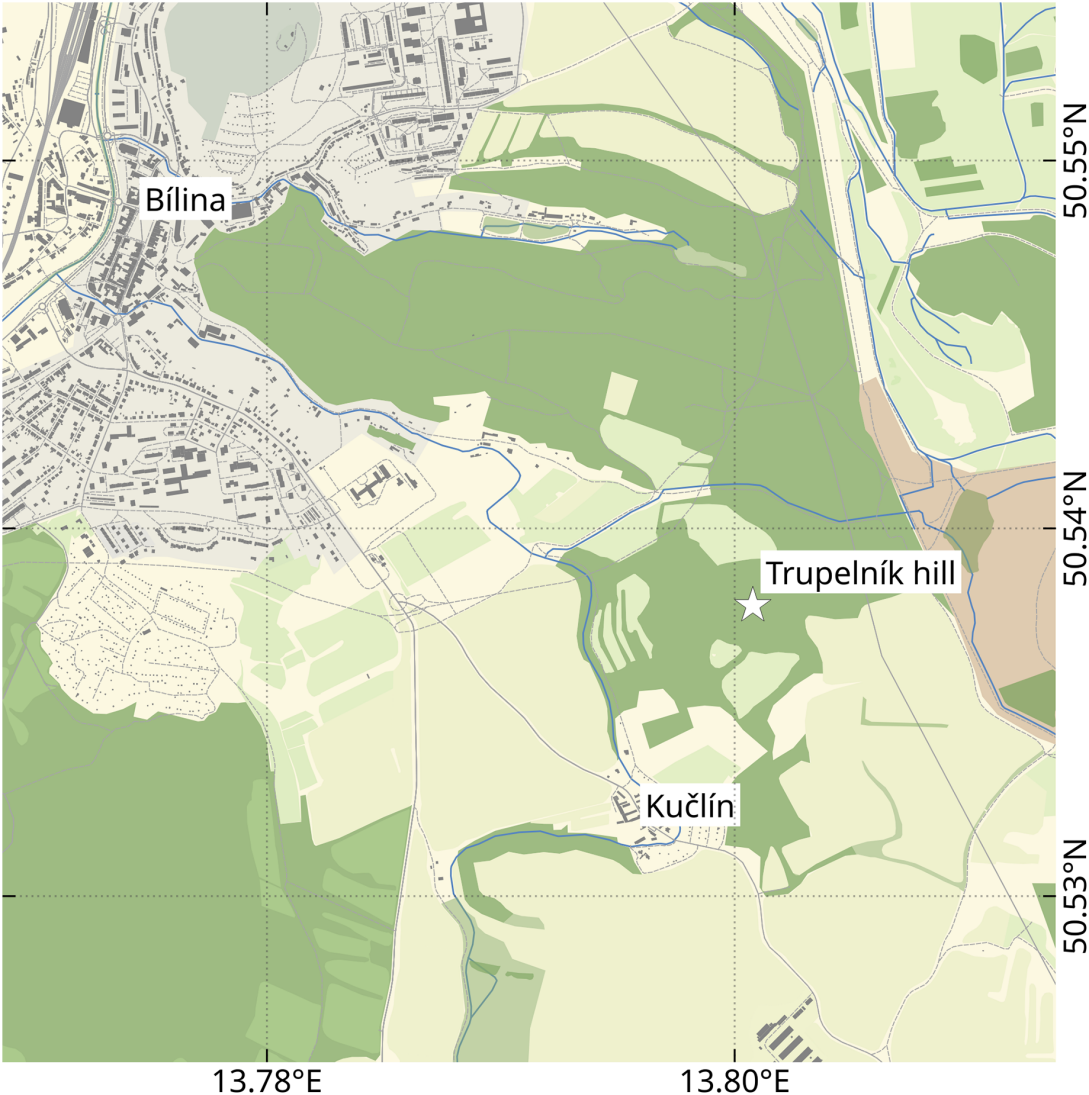
Location of the Trupelník hill field site (denoted by a white star), southeast of the town Bílina (Teplice District) and northwest of the village Kučlín, Northwestern Bohemia, Czech Republic. Map data from OpenStreetMap (openstreetmap.org, ODbL license).

**Figure 2:**
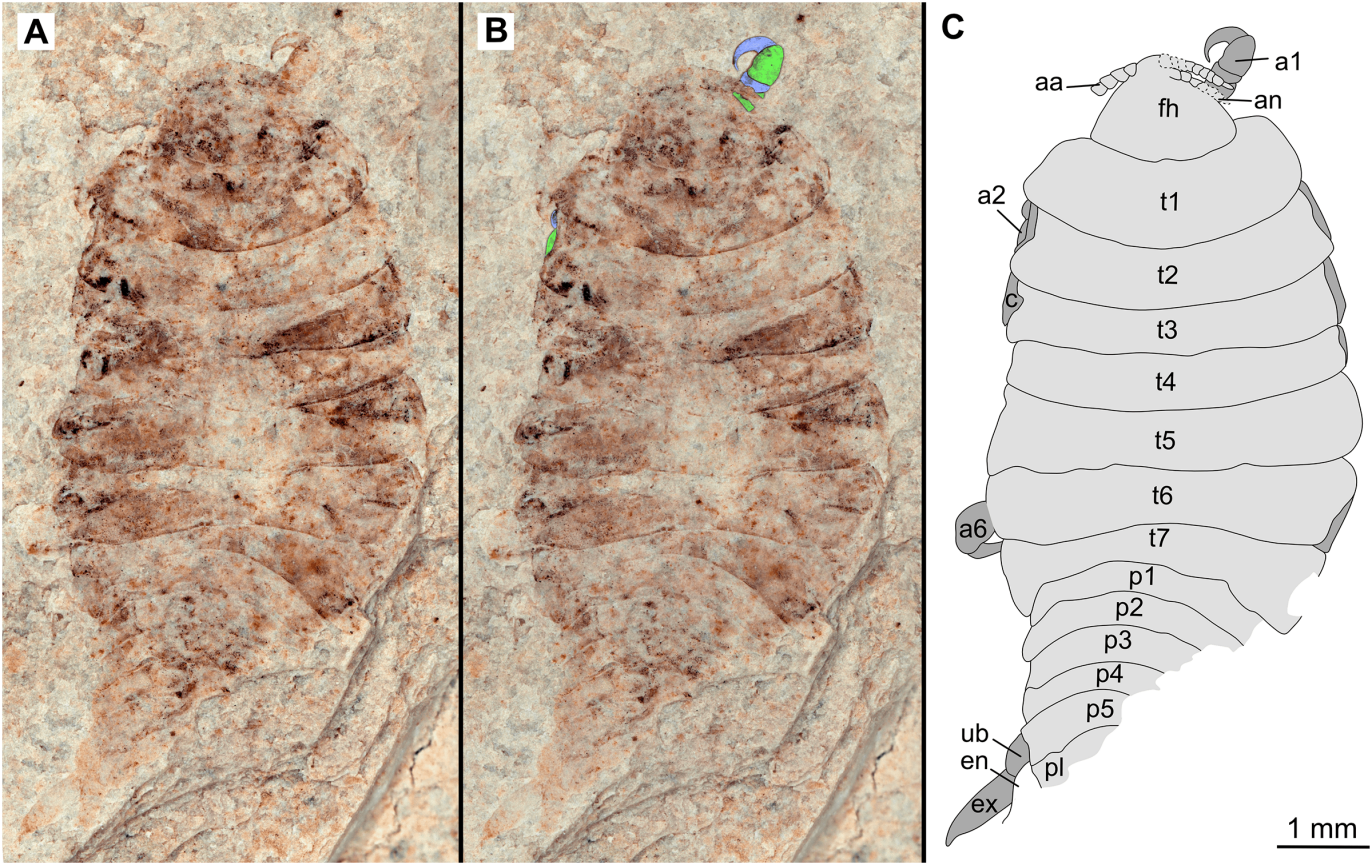
Holotype male (P2339a). (A–C) same scale. (A) Light microscope image with dorsal features and structures visible. (B) With colour marked trunk appendages, (C) Line drawing. Abbreviations: a1–2, trunk appendages 1–2; aa, antennula; ex, uropod exopod; fh, functional head; p1–5, pleon segments 1–5; pl, pleotelson; t1–7, trunk segments 1–7; ub, uropod basipod.

**Figure 3:**
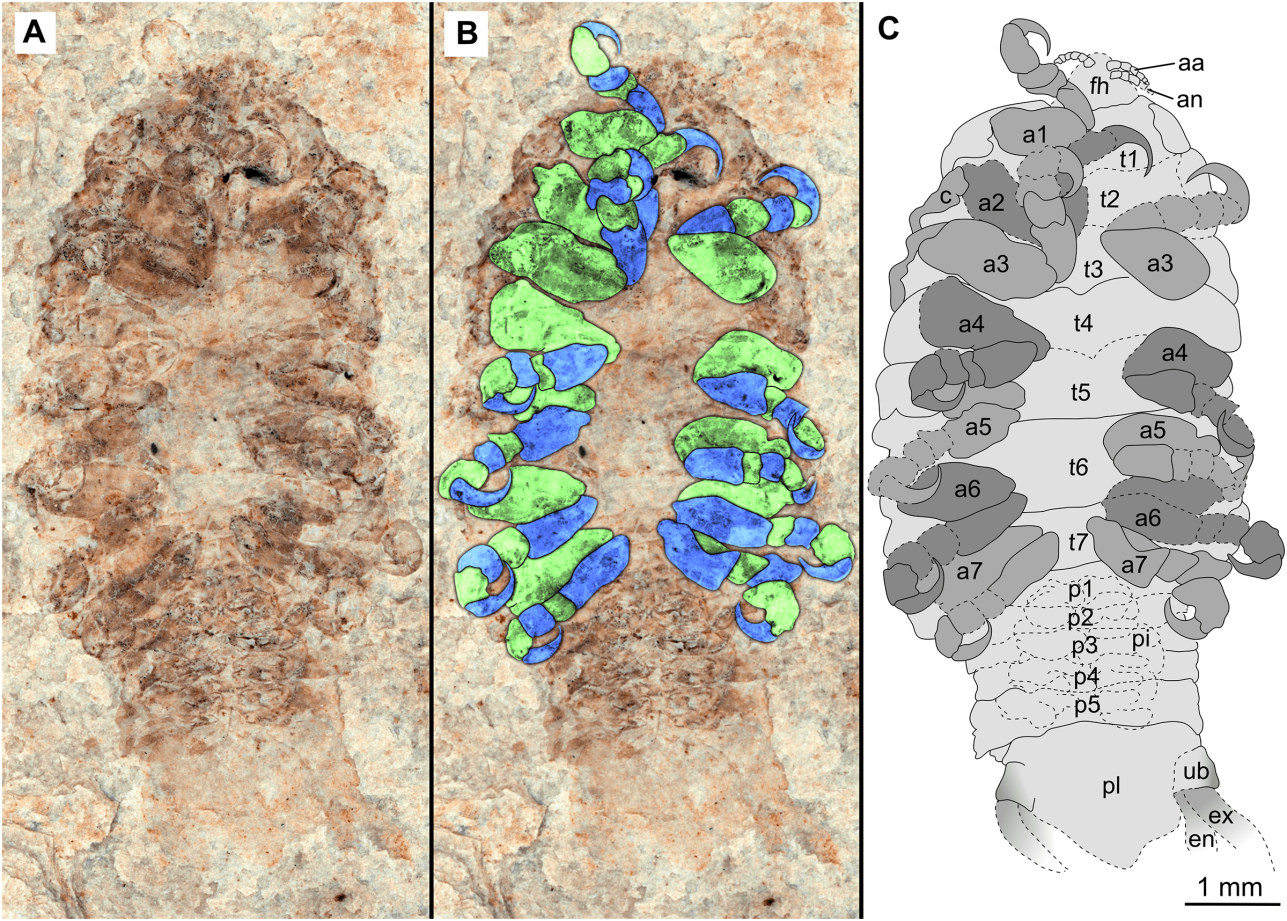
Holotype male (P2339b). (A–C) same scale. (A) Light microscope image with ventral features and structures visible. (B) With colour marked trunk appendages, (C) Line drawing. Abbreviations: a1–7, trunk appendages 1–7; aa, antennula; an, antenna; c, coxa; en, uropod endopod; ex, uropod exopod; fh, functional head; p1–5, pleon segments 1–5; pi, pleon attachment; pl, pleotelson; t1–7, trunk segments 1–7; ub, uropod basipod.

**Figure 4:**
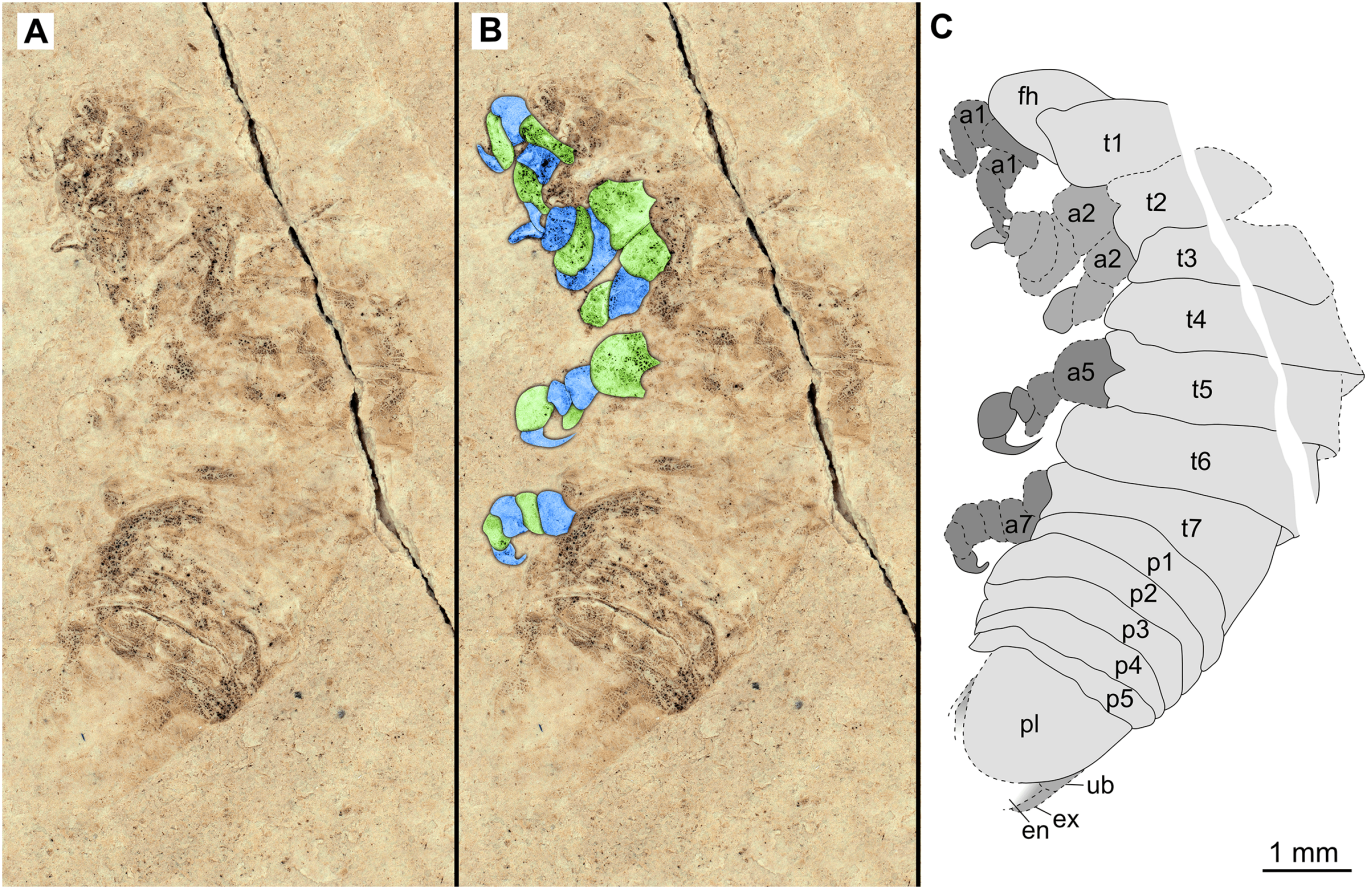
Specimen P2342a. (A–C) Same scale. (A) Light microscope image with dorso-lateral features and structures visible. (B) With colour marked trunk appendages. (C) Line drawing. Abbreviations: a1–2, trunk appendages 1–2; a5, trunk appendage 5; a7, trunk appendage 7; en, uropod endopod; ex, uropod exopod; fh, functional head; p1–5, pleon segments 1–5; pl, pleotelson; t1–7, trunk segments 1–7; ub, uropod basipod.

**Figure 5:**
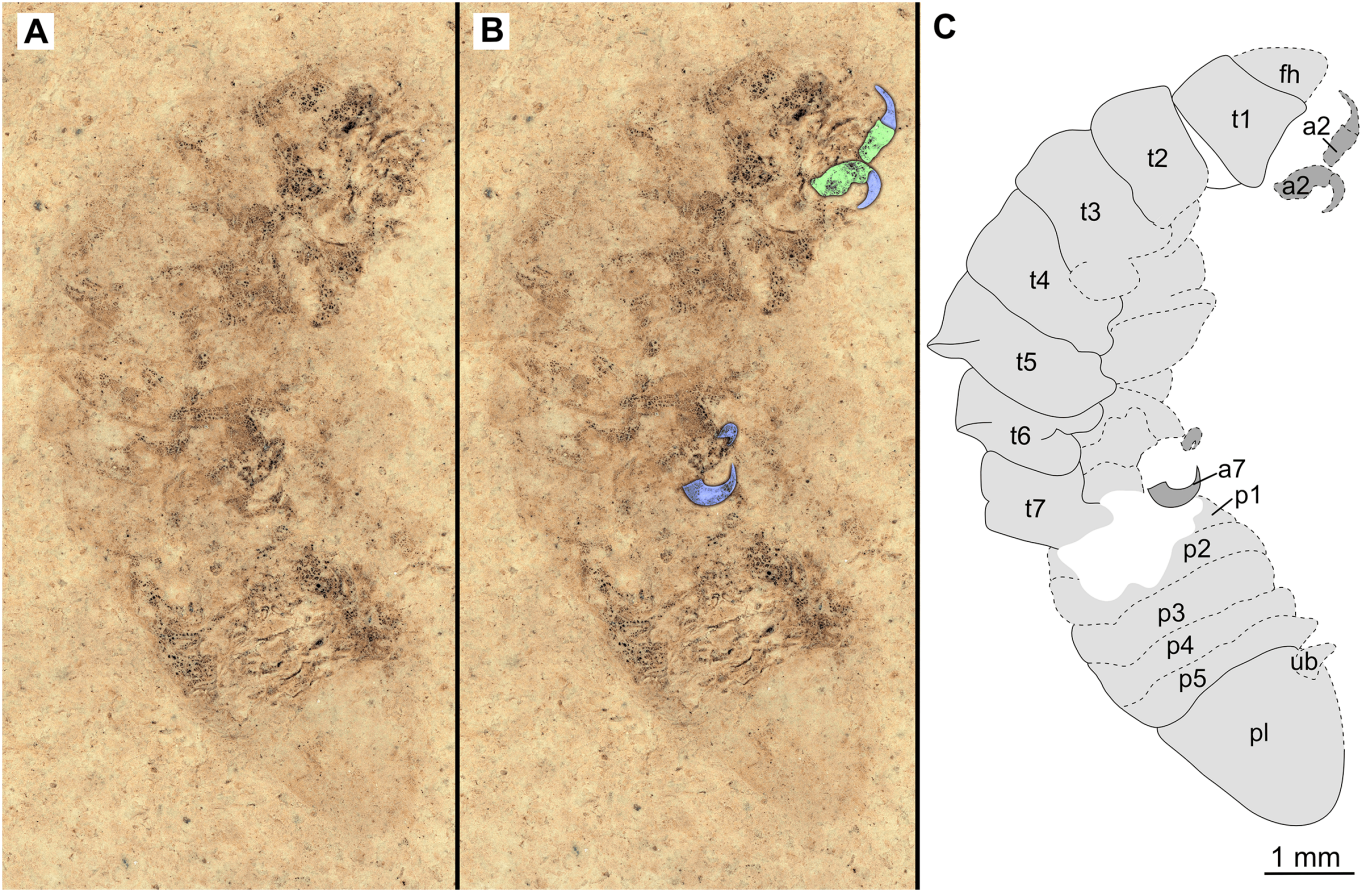
Specimen P2342b. (A–C) Same scale. (A) Light microscope image with dorso-lateral features and structures visible. (B) with colour marked trunk appendages. (C) Line drawing. Abbreviations: a2, trunk appendage 2; a7, trunk appendage 7; en, uropod endopod; ex, uropod exopod; fh, functional head; p1–5, pleon segments 1–5; pl, pleotelson; t1–7, trunk segments 1–7; ub, uropod basipod.

**Figure 6:**
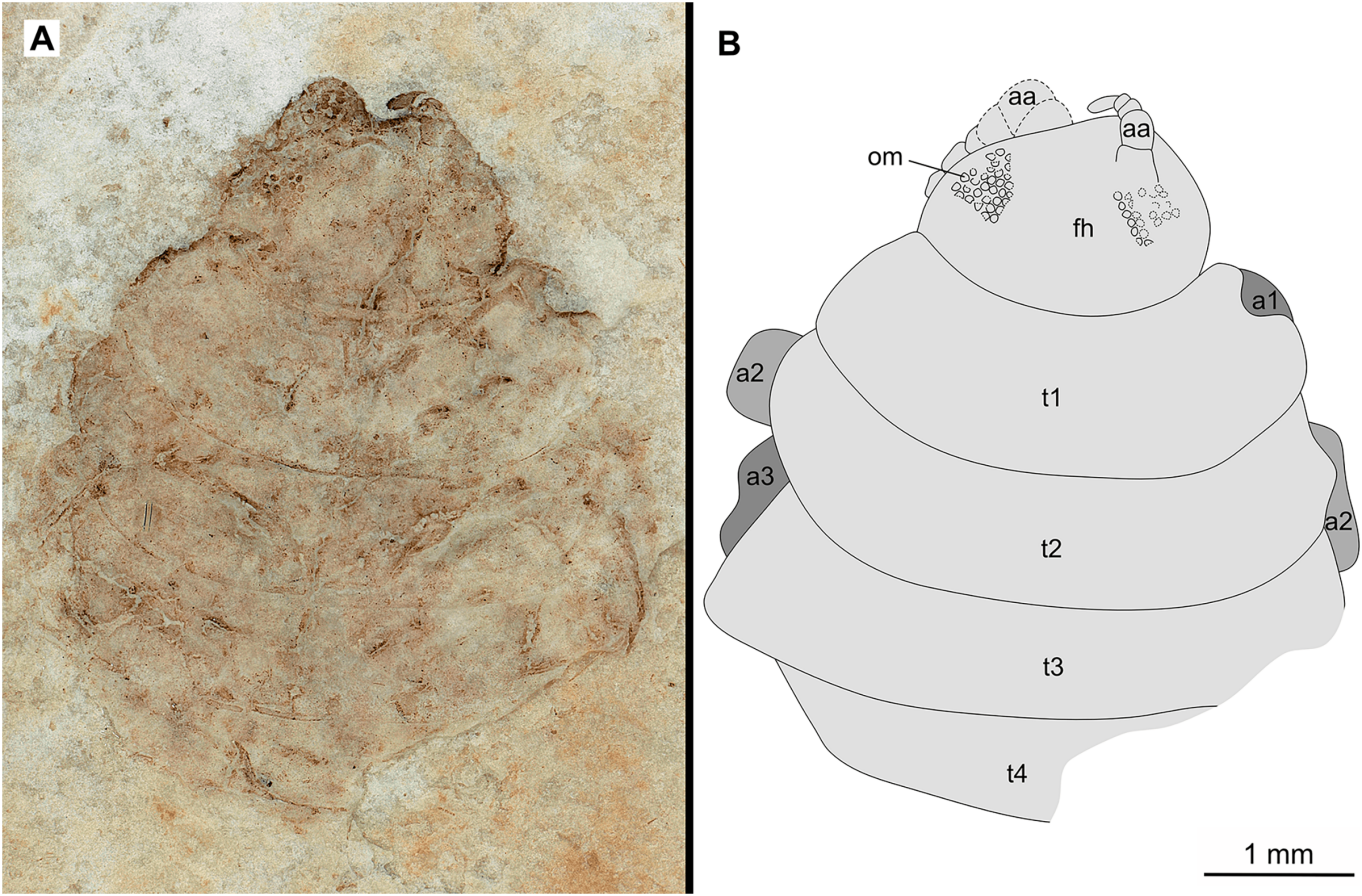
Specimen P2346a. (A–B) Same scale. (A) Light microscope image with dorsal features and structures visible. (B) Line drawing. Abbreviations: a1–3, trunk appendages 1–3; aa, antennula; om, ommatidium of compound eye; fh, functional head; t1–4, trunk segments 1–4.

**Figure 7:**
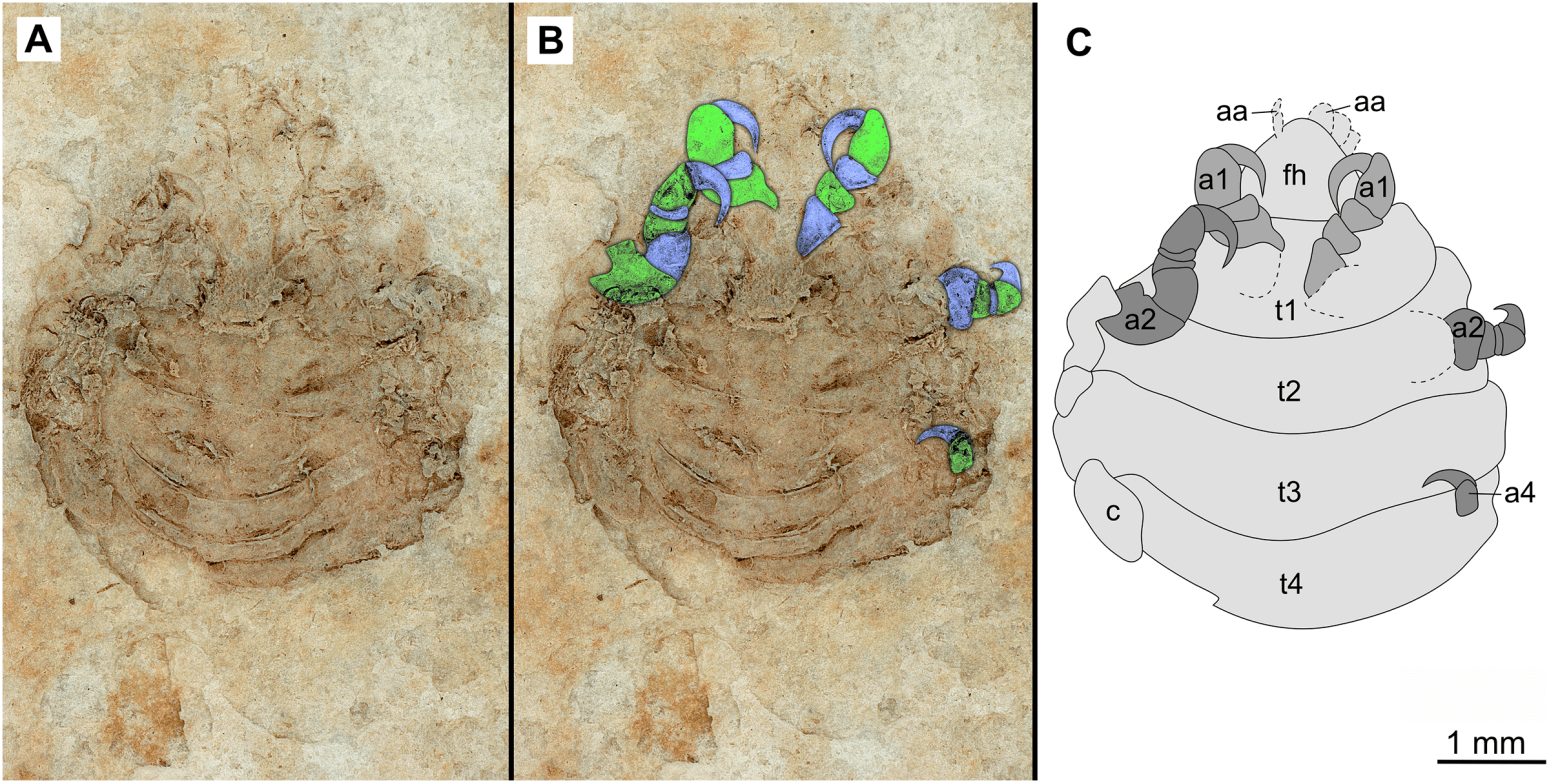
Specimen P2346b. (A–C) Same scale. (A) Light microscope image with ventral features and structures visible. (B) With colour marked trunk appendages. (C) Line drawing. Abbreviations: a1–2, trunk appendages 1–2; a4, trunk appendage 4; aa, antennula; c, coxa; fh, functional head; t1–4, trunk segments 1–4.

**Figure 8:**
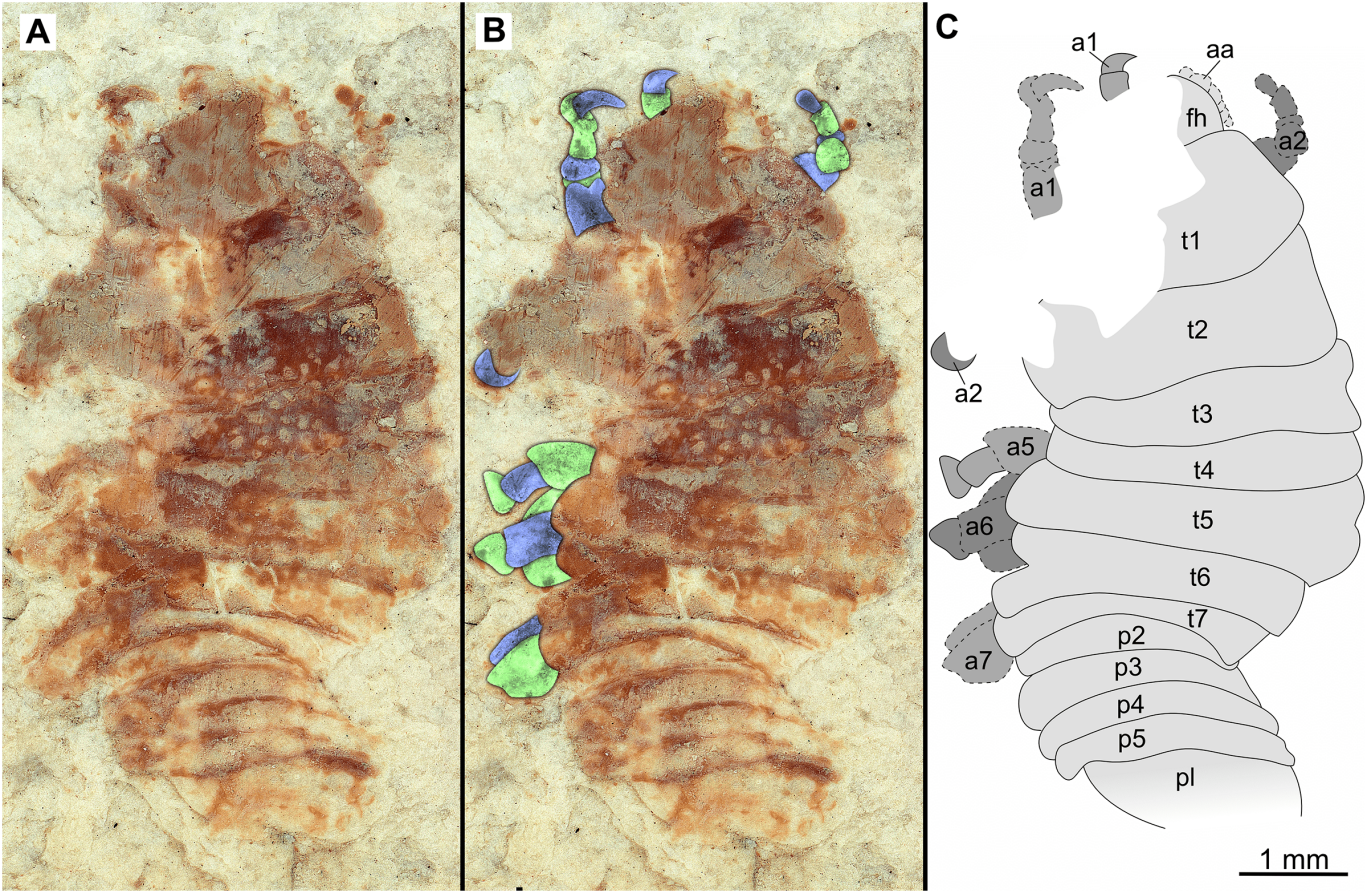
Specimen P2348. (A–C) Same scale. (A) Light microscope image with dorso-lateral features and structures visible. (B) With colour marked trunk appendages. (C) Line drawing. Abbreviations: a1–2, trunk appendages 1–2; a5–7, trunk appendages 5–7; aa, antennula; fh, functional head; p1–5, pleon segments 1–5; pl, pleotelson; t1–7, trunk segments 1–7.

**Figure 9:**
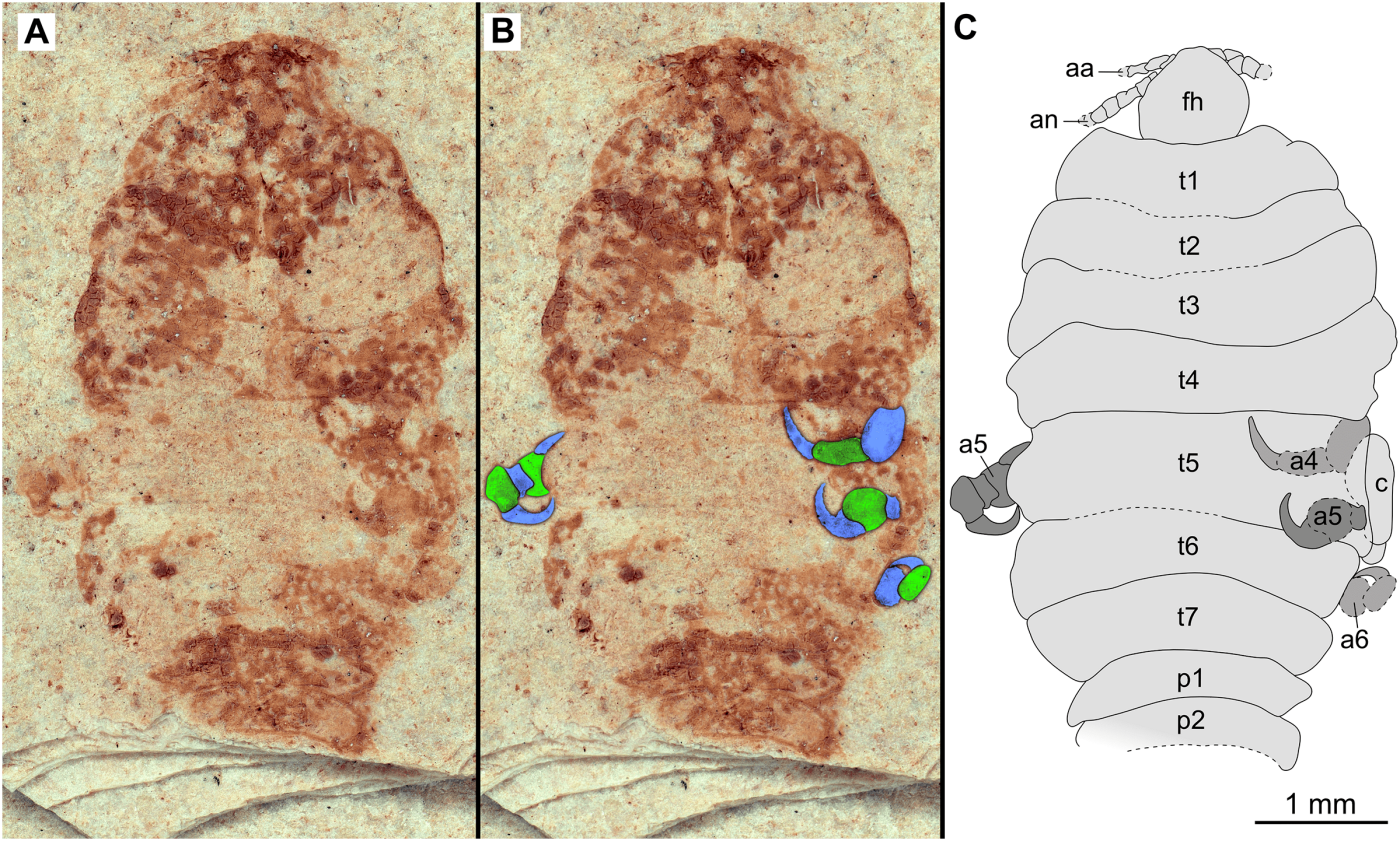
Paratype immature (P2338a). (A–C) Same scale. (A) Light microscope image with dorsal features and structures visible. (B) With colour marked trunk appendages. (C) Line drawing. Abbreviations: a4–6, trunk appendages 4–6; aa, antennula; an, antenna; c, coxa; fh, functional head; p1–2, pleon segments 1–2; t1–7, trunk segments 1–7.

**Figure 10:**
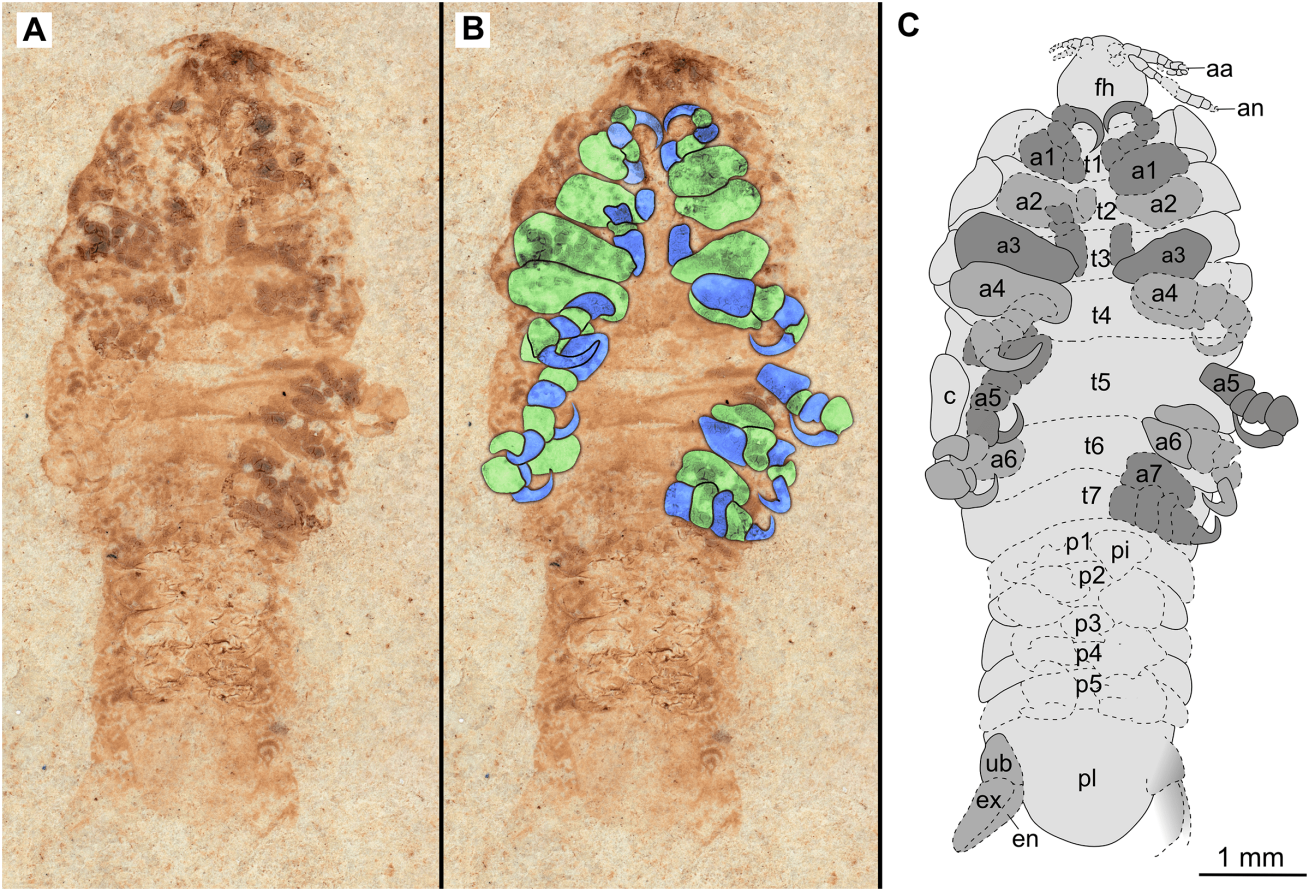
Paratype immature (P2338b). (A–C) Same scale. (A) Light microscope image with ventral features and structures visible. (B) With colour marked trunk appendages. (C) Line drawing. Abbreviations: a1–7, trunk appendages 1–7; aa, antennula; an, antenna; c, coxa; en, uropod endopod; ex, uropod exopod; fh, functional head; p1–5, pleon segments 1–5; pi, pleon attachment; pl, pleotelson; t1–7, trunk segments 1–7; ub, uropod basipod.

**Figure 11:**
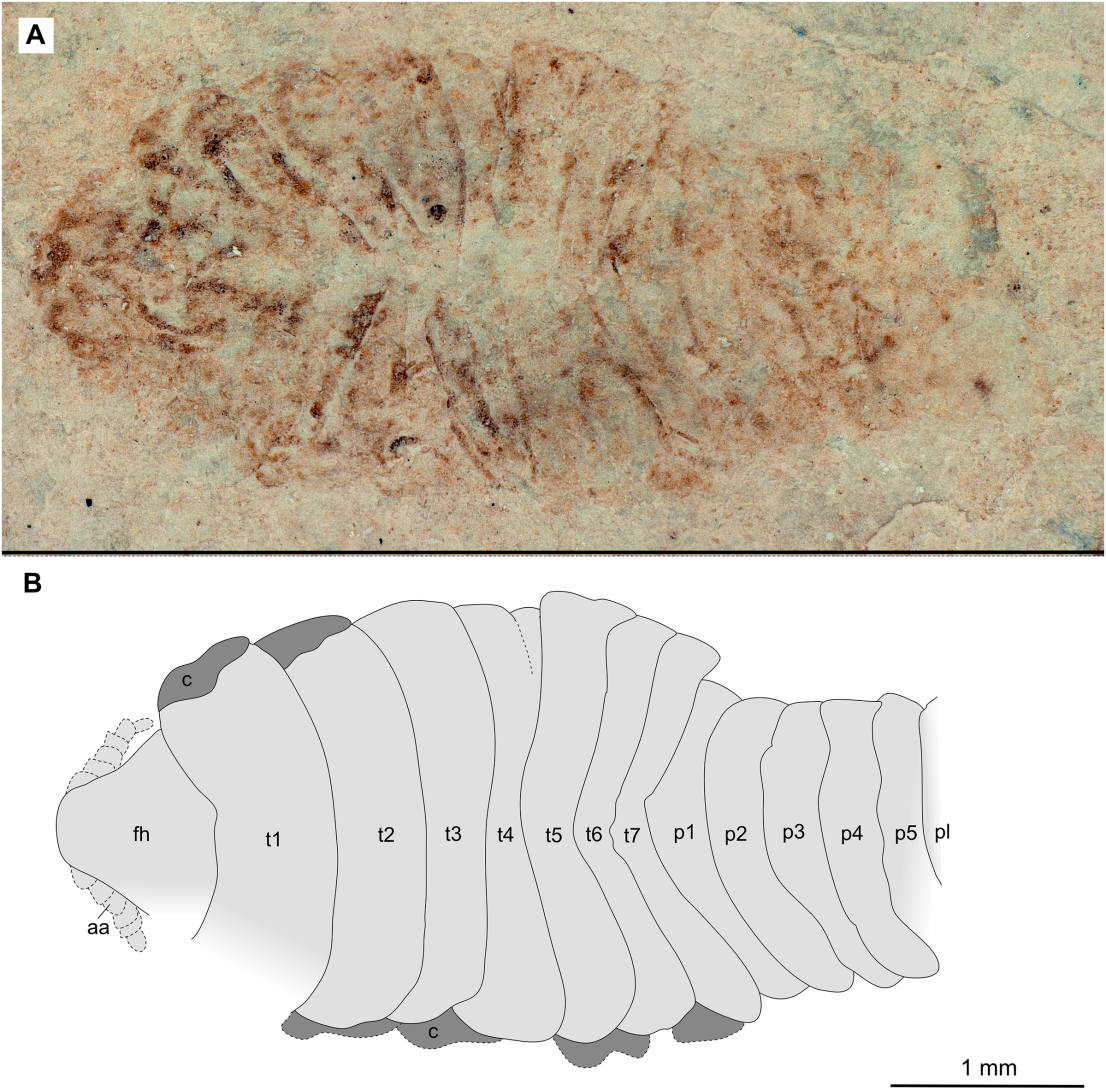
Specimen P2347. (A–B) Same scale. (A) Light microscope image with dorsal features and structures visible. (B) Line drawing. Abbreviations: a7, trunk appendage 7; aa, antennula; c, coxa; fh, functional head; p1–5, pleon segments 1–5; pl, pleotelson; t1–7, trunk segments 1–7.

**Figure 12:**
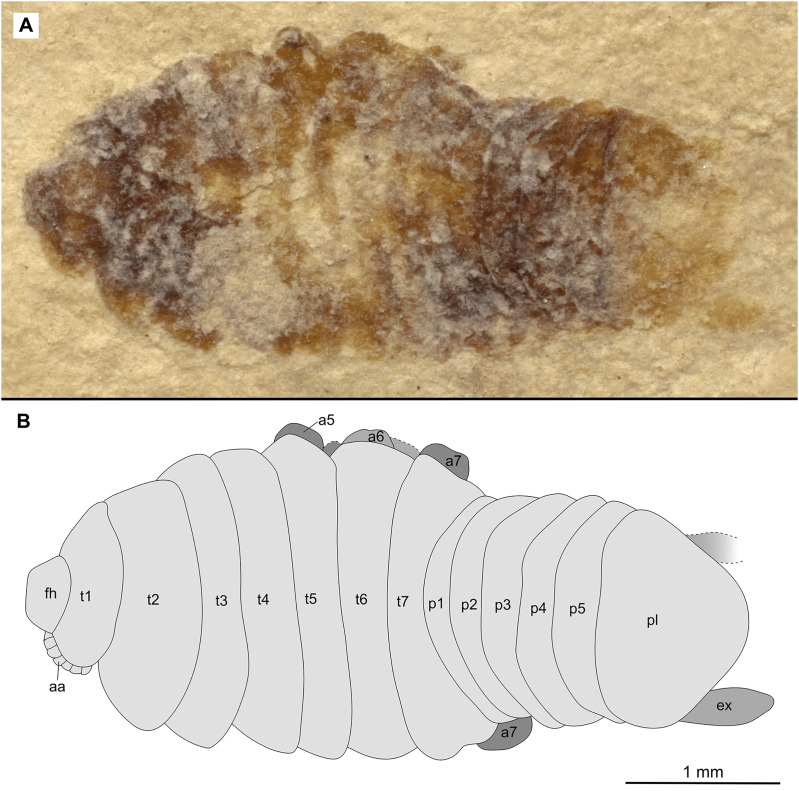
Specimen P2344. (A–B) Same scale. (A) Light microscope image with dorsal features and structures visible. (B) Line drawing. Abbreviations: a7, trunk appendage 7; aa, antennula; ex, uropod exopod; fh, functional head; p1–5, pleon segments 1–5; pl, pleotelson; t1–7, trunk segments 1–7.

**Figure 13:**
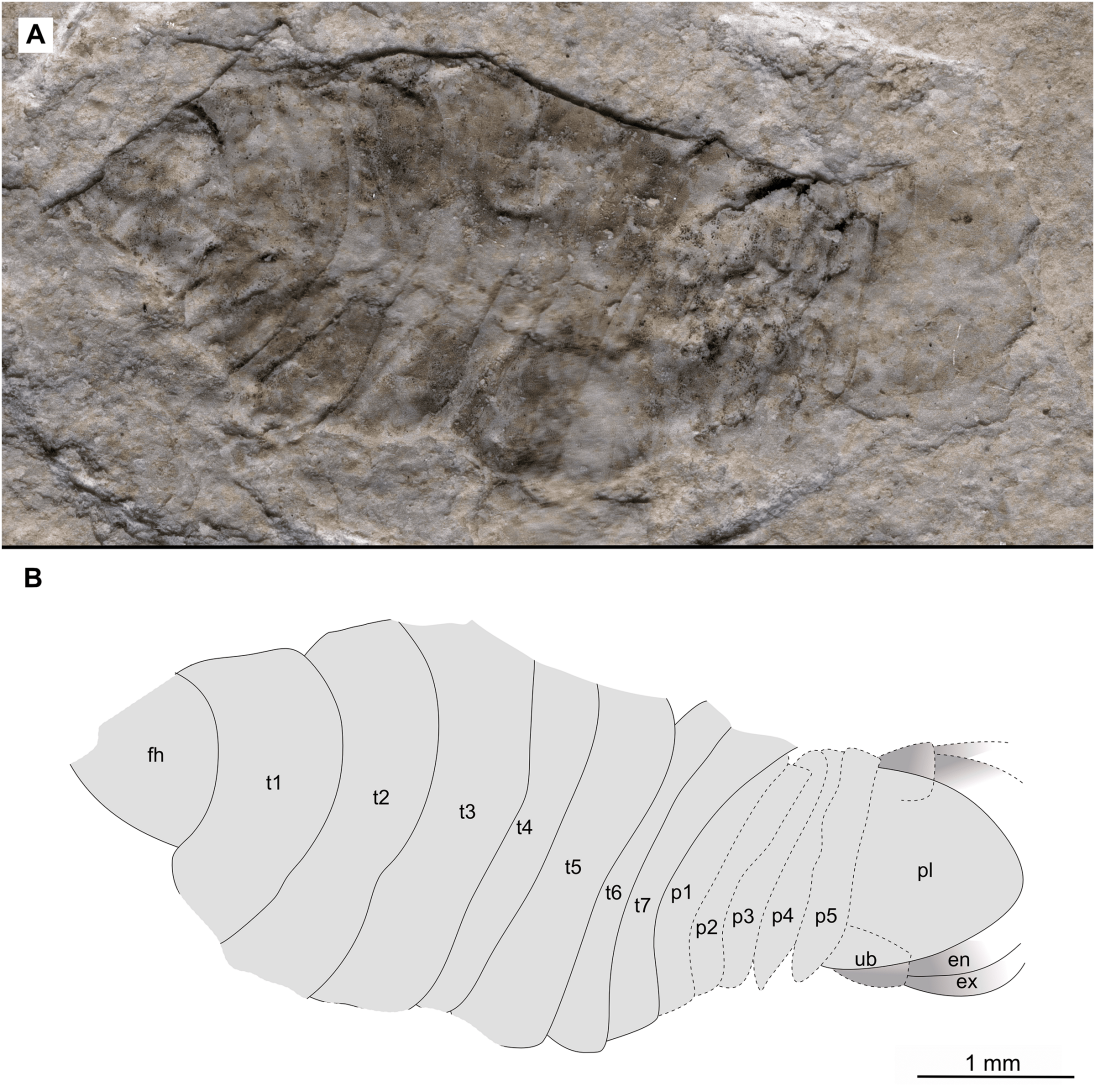
Specimen P2343. (A–B) Same scale. (A) Light microscope image with dorsal features and structures visible. (B) Line drawing. Abbreviations: en, uropod endopod; ex, uropod exopod; fh, functional head; p1–5, pleon segments 1–5; pl, pleotelson; t1–7, trunk segments 1–7; ub, uropod basipod.

**Figure 14:**
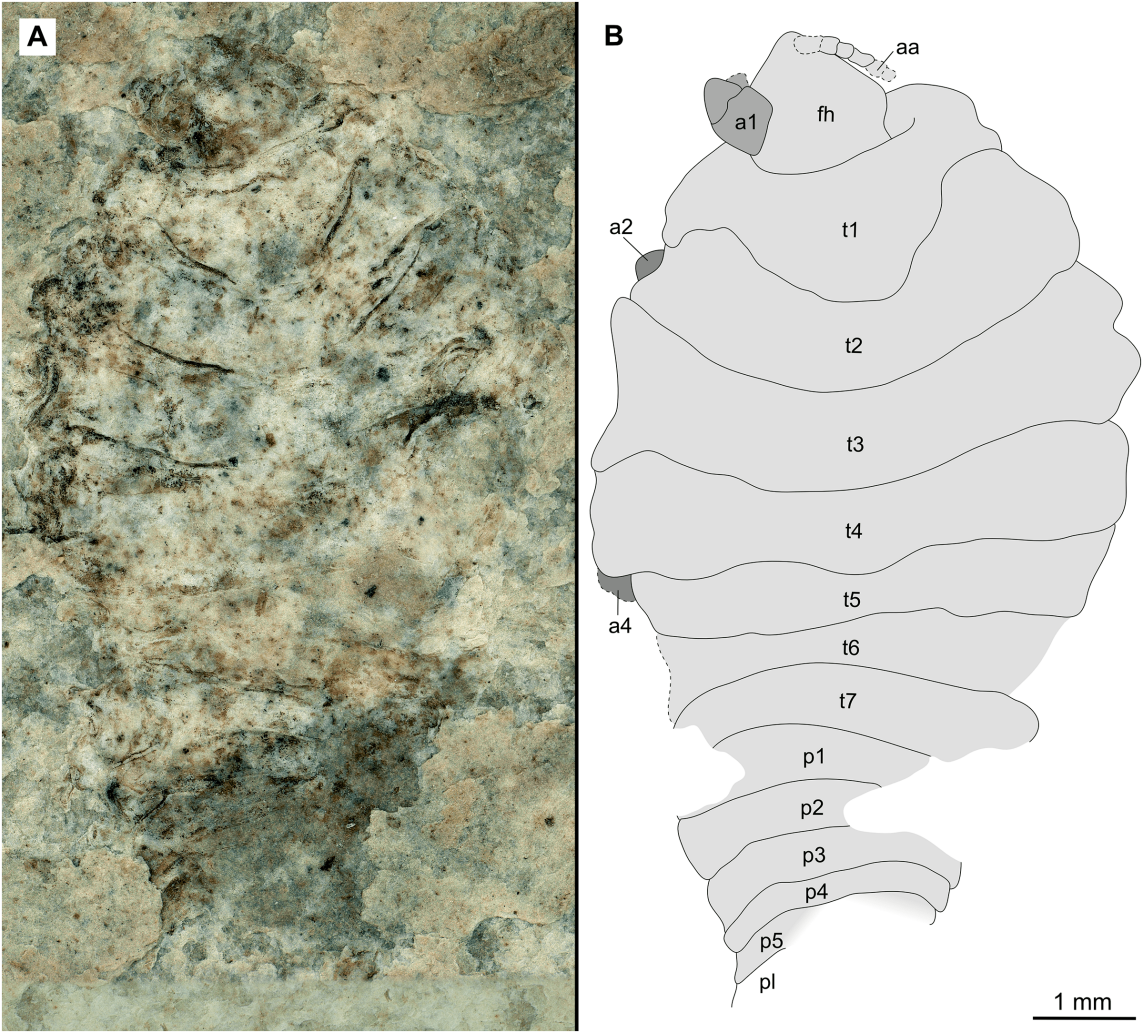
Paratype female (P2345a). (A–B) Same scale. (A) Light microscope image with dorsal features and structures visible. (B) Line drawing. Abbreviations: a1–2, trunk appendages 1–2; a4, trunk appendage 4; aa, antennula; fh, functional head; p1–5, pleon segments 1–5; pl, pleotelson; t1–7, trunk segments 1–7.

**Figure 15:**
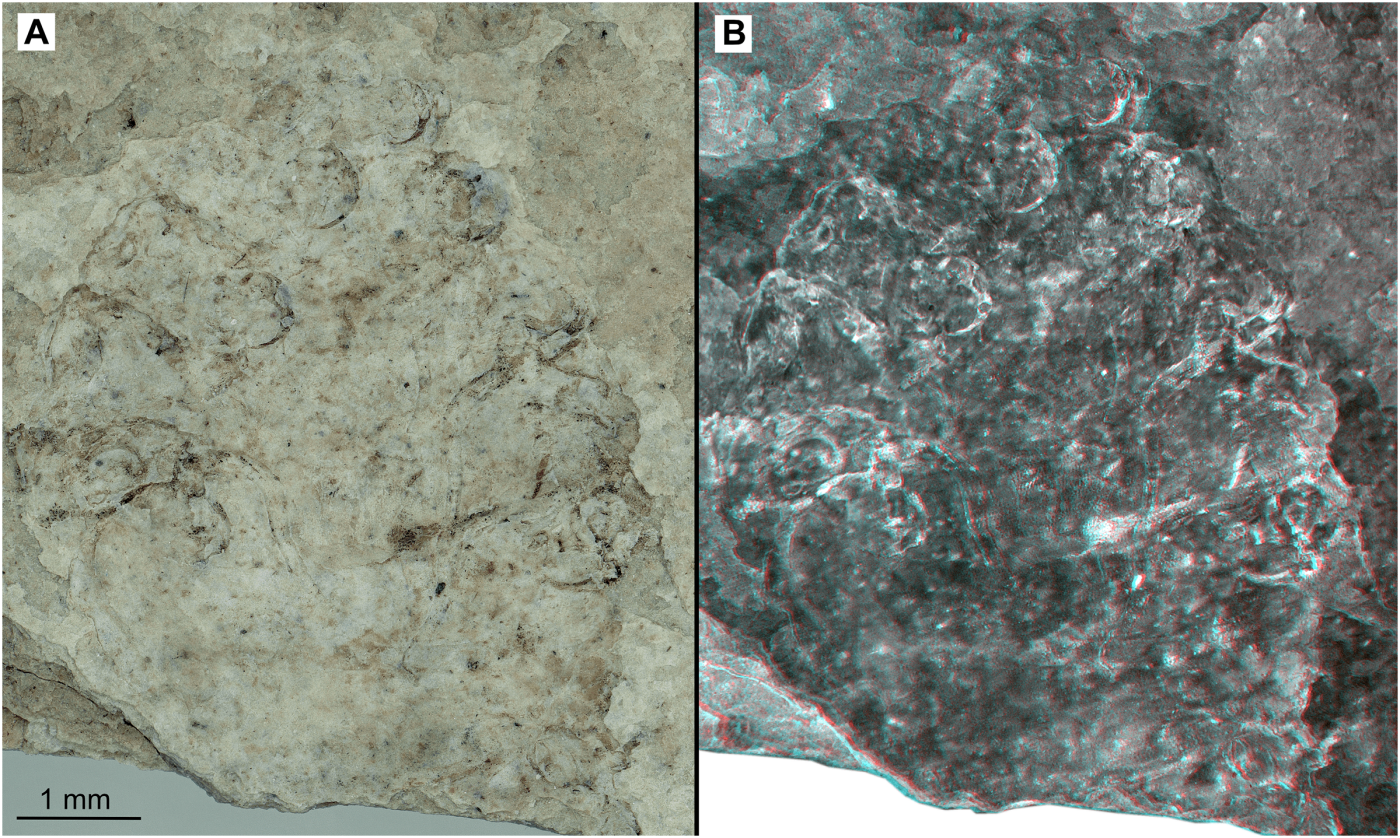
Paratype female (P2345b). (A–B) Same scale. (A) Light microscope image with ventral features and structures visible. (B) Three dimentional stereo-photograph.

**Figure 16:**
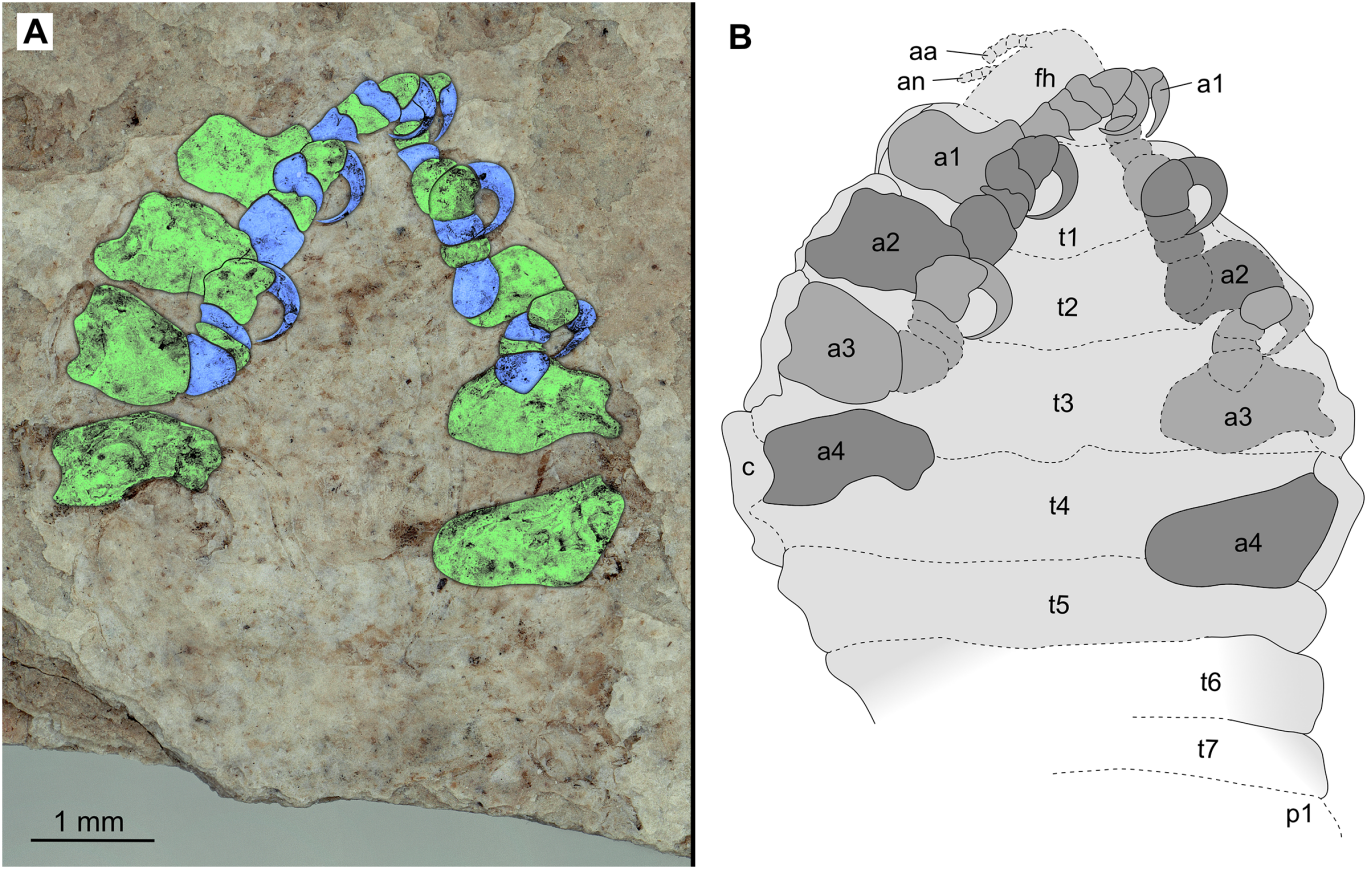
Paratype female (P2345b). (A–B) Same scale. (A) Light microscope image with ventral features and structures visible. (B) Line drawing. Abbreviations: a1–4, trunk appendages 1–4; aa, antennula; an, antenna; c, coxa; fh, functional head; p1, pleon segment 1; t1–7, trunk segments 1–7.

**Figure 17:**
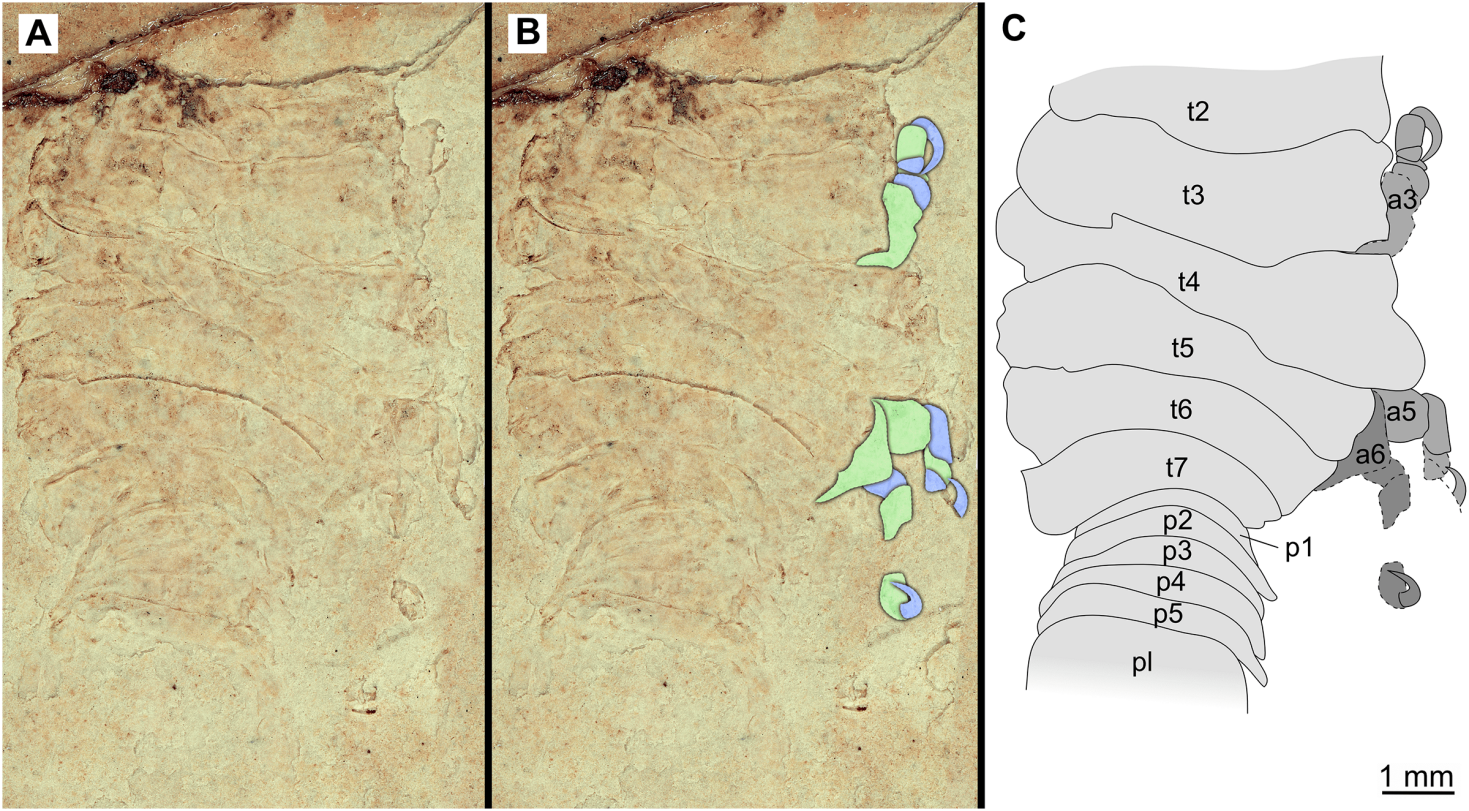
Specimen P2341. (A–B) Same scale. (A) Light microscope image with dorsal features and structures visible. (B) With colour marked trunk appendages. (C) Line drawing. Abbreviations: a3, trunk appendage 3; a5, trunk appendage 5; a6, trunk appendage 6; p1–5, pleon segments 1–5; pl, pleotelson; t2–7, trunk segment 2–7.

**Figure 18:**
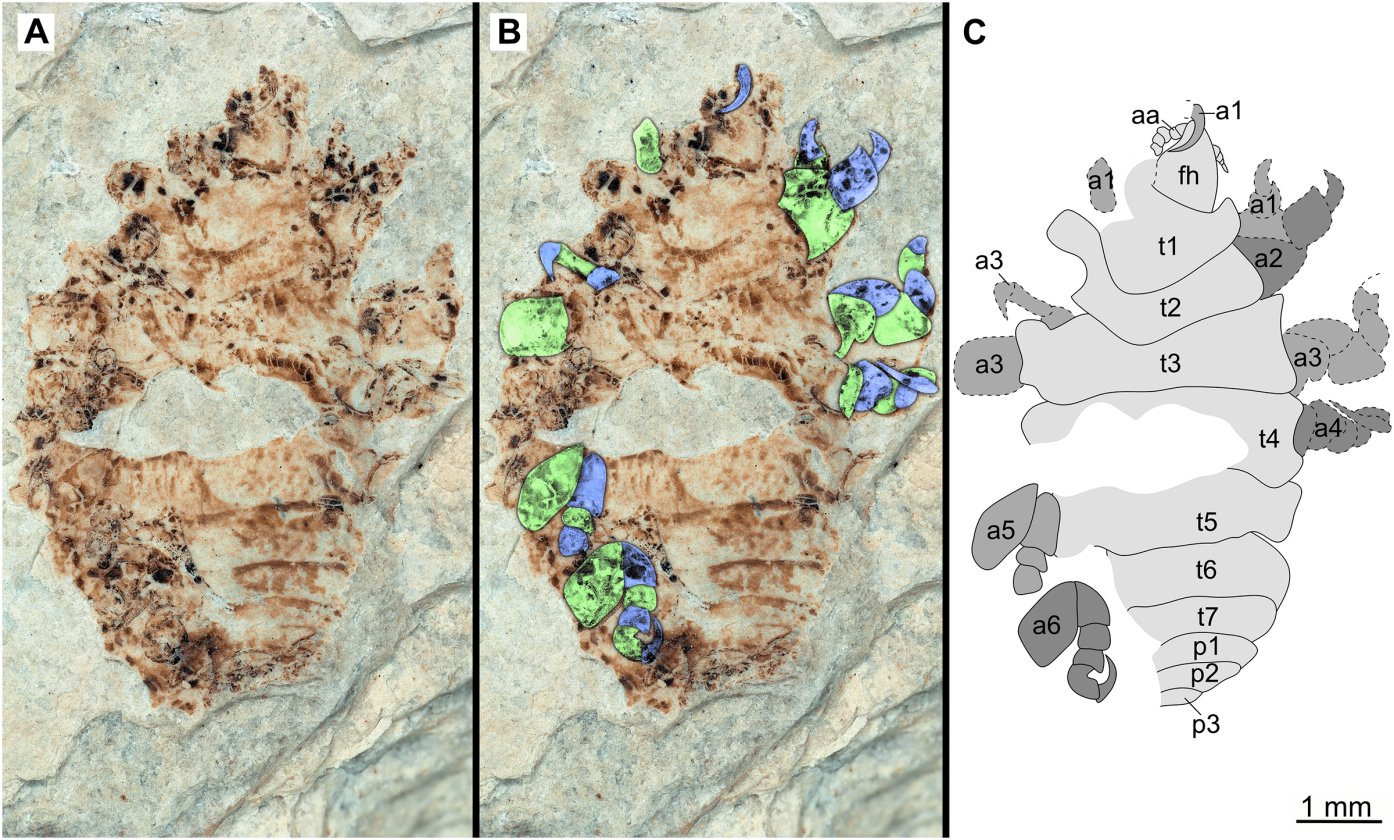
Specimen P2340. (A–B) Same scale. (A) Light microscope image with dorsal features and structures visible. (B) With colour marked trunk appendages (C) Line drawing. Abbreviations: a1–6, trunk appendage 1–6; aa, antennula; fh, functional head; p1–3, pleon segments 1–3; t1–7, trunk segments 1–7.

The electronic version of this article in Portable Document Format (PDF) will represent a published work according to the International Commission on Zoological Nomenclature (ICZN), and hence the new names contained in the electronic version are effectively published under that Code from the electronic edition alone. This published work and the nomenclatural acts it contains have been registered in ZooBank, the online registration system for the ICZN. The ZooBank LSIDs (Life Science Identifiers) can be resolved and the associated information viewed through any standard web browser by appending the LSID to the prefix http://zoobank.org/. The LSID for this publication is: urn:lsid:zoobank.org:pub:C38FC926-EEC4-45F8-8CBB-3639D845C4DA. The online version of this work is archived and available from the following digital repositories: PeerJ, PubMed Central and CLOCKSS.

### Geological setting and palaeoenvironment

The herein presented fossils come from the so called ‘upper pothole quarry’ of the Trupelník hill field site, near Kučlín (České středohoří mountain range, North Bohemia, Czech Republic; see Fig. 1). This fossil site was first mentioned in publications at the end of the 18th century and throughout the 19th–21st centuries. It afforded rich palaeontological material. Private and particularly commercial collecting was focused mostly on decorative fish skeletons, plant particles and sometimes certain insects. Small, non-decorative fossils, such as those presented herein, have usually been neglected. Comprehensive collecting was done by Zdeněk and Pavel Dvořák over the last 25 years.

The sediments in which the fossils were found are late Eocene in age (see Fejfar & Kvaček, 1993). Basaloid rock (sodalite tephrite) that overlie the sediments have been dated to an age of 38.3 ± 0.9 million years (Bellon et al., 1998). Subsequently, the sedimentary rocks below this, including the herein presented fossils, are only slightly older. The late Eocene age of the sedimentary rocks, which contain the fossils herein presented, is also corroborated *via* biostratigraphy of pollen of *Compositoipollenites rhizophorus* (R.Pot., 1934) R. Pot., 1960 and *Striatricolpites catatumbus* Gonzalez, 1967 (Konzalová, 1981).

The fossils were excavated from finely laminated diatomites. The exact composition of the rock matrix and the degree of compaction and diagenesis between the individual layers of sediment, varies considerably. The sediments were most likely deposited in a freshwater lake within a geological basin (Mach & Dvořák, 2011). Even though there is no geological indication for a connection of the depositional environment with the ocean (Mach & Dvořák, 2011), such a connection can be suggested by the presence of temperate basses (Moronidae). These fish have been assumed to have populated the environment *via* a river system or that they represent primarily marine animals with anadromous behaviour (Micklich, 1990; Micklich & Böhme, 1997; Přikryl, 2008). Except for the relatively rare representatives of *Morone*, three abundant species of ray-finned fishes have been collected from this site (*Properca prisca* (Agassiz, 1834); *Thaumaturus furcatus* Reuss, 1844; *Cyclurus macrocephalus* Reuss, 1844). The presence of possible parasites, *in situ*, was carefully checked for all of the collected fish fossils, but none were found. Parasitic representatives of Isopoda can easily be overlooked during the preparation of a fossil, especially since re-crystallisation of the crustacean can appear as an insignificant crystalline blob (Nagler et al., 2016). The most likely connection to the ocean would have been towards the north into the Atlantic Ocean (Micklich & Böhme, 1997; Scotese, 2014). Palaeoclimate reconstructions, based on the fossil flora and fauna of the Trupelník Hill field site, suggest a seasonal warm-temperate to subtropical palaeoenvironment during the late Eocene (Kvaček, 2002; Kvaček & Teodoridis, 2011; Chroust, Mazuch & Hernández Luján, 2019).

### Documentation methods

Fossil specimens were photographed under white light using a Keyence VHX-6000 digital microscope. The built-in focus fusion technique of the digital microscope was used to achieve full focus images. Stereo images were created by tilting the microscope seven degrees to the left and to the right, respectively, and recording full focus images (Wheatstone, 1838). The stereo images were converted into red-cyan stereo anaglyphs (Rollmann, 1853) using Affinity Photo (Serif Europe Ltd). In case the stereo anaglyphs cannot be perceived by the reader, they can be converted into wiggle images using free software such as kataglyph (GPL licence, available from https://github.com/mcranium/kataglyph). Image editing and enhancement was done using Affinity Photo. Line drawings, colour markings of body parts, and assembly of figure plates were prepared using a combination of Adobe Illustrator (Adobe Inc.) and Affinity Designer. All drawings are available from the ‘MorphDBase’ online repository *via* the permanent link www.morphdbase.de/. Exact links to the figures of each respective specimen are provided in *Material examined*.

### Field site map

The map depicting the location of the ‘Trupelník hill’ field site was created using QGIS v.3.14 (qgis.org, GPL license). The map data comes from OpenStreetMap (openstreetmap.org, ODbL licence) and was retrieved using the QuickOSM plugin for QGIS (GPL v.2 licence).

### Terminology

Specialised terminology often prohibit communication beyond a specific taxonomic border. In order to avoid the confusion regarding terms used for specific structures, these are provided here. Descriptions comprise terminology used for the general Eumalacostraca body organisation and articulation (based on Walossek, 1999) which can be compared to Isopoda specific terms as used by for example Jackson (1926), Kensley (1978) and Hoffman (2019). A further comparison between preferred terms among isopod- and other crustacean workers is provided in Nagler, Eiler & Haug (2019). The descriptions herein comprise the following terminology: a functional head (in literature also referred to as cephalon or cephalothorax), bearing the ocular segment and six post-ocular segments, including the corresponding appendages (antennula, antenna, mandible, maxillula, maxilla and maxilliped); an anterior trunk (in literature also referred to as the posterior thorax or pereon) of seven segments (thoracomeres, also referred to as pereonites), each with one pair of appendages (thoracopods, also referred to as pereopods); a posterior trunk (pleon) comprising five anterior segments (pleomeres, or also pleonites), each with one pair of appendages (pleopods) and the sixth pleon segment conjoined to the telson forming the pleotelson, with one pair of appendages (uropods). Additionally, species of the group Cymothoidae are protandric, meaning that a “male” will eventually develop into a female and is therefore regarded as a separate ontogenetic stage.

### Measurements, descriptions and morphometrics

Measurements of the examined fossils include the following distances, measured using ImageJ (public domain): The total length and width of the complete specimen, where completely preserved; maximum length and maximum width of the head, each completely preserved anterior trunk segment, each completely preserved element of trunk appendages, each completely preserved pleon segment, and pleotelson (where preserved). These measurements were used to calculate ratios of the completely preserved structures, used in the descriptions. Only structures that were complete and preserved without distortion, were measured (in mm) to avoid inaccuracy due to perspective. Measurements were rounded to two decimal points, ratios were rounded to one decimal point. Specimen descriptions were made with structures in the direction from anterior to posterior and from proximal to distal.

A comparative overall body outline analysis was done using: (1) the reconstructed illustrations of examined specimens from which a complete and undistorted dorsal side was preserved; (2) and those of different ontogenetic stages of various extant species. This provided information on the variation in body shape between the examined fossils, among the examined fossils and extant species, as well as between different ontogenetic stages.

From literature, the body outlines of 18 extant species (dorsoventral projection) were included in the analysis. The selection of species was made based on: (1) the availability of dorsal view illustrations or photographs of at least three different ontogenetic stages of a species (*i.e.*, female, male and immature stage), and (2) the site of attachment (*i.e.*, mouth, gill and externally attaching parasitic groups). A total of 76 individual outlines were included in the analysis, along with five reconstructed outlines of completely preserved examined fossils.

The reconstructions were done manually with the aid of the software program Affinity Designer. Interpretive digital illustrations were made of specimens P2338, P2339, P2347, P2344 and P234 as these specimens have the best preservation in terms of orientation (accessible in dorsal view) in order to avoid or reduce the degree of idealisation when creating reconstructions. From the fossils it is evident that the specimens had a bilaterally symmetrical body, which was used as a guideline for reconstruction. Undistorted body segments were arranged and distorted segments symmetrized (idealised) in a way that would provide a complete and smoother body outline, with minimum alteration in the shape and proportions of the segment. For this, the best preserved lateral side of a segment was chosen to serve as a guide. This side (left or right from the medial symmetry line of the specimen) was then mirrored on the opposite side to create a complete segment which is bilaterally symmetrical.

For the list of species included and publications from which the additional illustrations were redrawn, see Doc. S1. Illustrations of curved specimens were straightened by deforming a vectorised copy of the outline in Inkscape (GPL-2 licence) using the ‘bend from clipboard’ function with a mirrored midline of the shape. ImageMagick (Apache 2.0 licence) was used for batch resizing and converting raster image files. The quantitative analysis of the outline shapes was performed using the R programming language (R Core Team, 2020, v.3.6.3). Momocs (GPL-3 licence; Bonhomme et al., 2014) was used to read the raster image files. The outlines were automatically centred, scaled and aligned using functions from the Momocs package. The ‘efourier’ function from Momocs was used to convert the shape information from a coordinate based format to Fourier coefficients (elliptic Fourier transformation). For this, 10 harmonics were used and the Fourier coefficients were automatically normalized. The Fourier coefficients were then ordinated using the Principle Component Analysis (PCA) function implemented in Momocs. Linear models (‘lm’ function, base R) were fitted to the first two principle components relative to the total body length.

Additional R packages were used for data manipulation (‘dplyr’, ‘magrittr’, ‘reshape2’) (Wickham, 2007; Bache & Wickham, 2014; Wickham et al., 2020). The web application ‘iWantHue’ (GPL-3 licence, https://medialab.github.io/iwanthue/) was used to choose colours used in the plots that are suitable for colour vision impaired persons. The colours were additionally checked, using the software Color Oracle 1.3 (CC-BY licence, Bernhard jenny and Nathaniel V. Kelso). The R code used for this analysis is available from Doc. S2.

The dataset imported to R, is given in Doc. S1, with the code created and applied for visualising the results as plots, given in Doc. S2. A total of 76 dorsal view body shapes were analysed together. To visualise the variation in the outline shapes and to simplify the data, a principal component analysis (PCA) was done. The variation in the principle components (PC1–PC10) is given in Fig. S1. The mean shapes of each ontogenetic stage (immature, male and female) are presented and compared in Fig. S2.

## Results

*Systematic palaeontology*

Cymothoida Wägele, 1989

Cymothoidae Leach, 1814

*Parvucymoides* gen. nov. ZooBank LSID: urn:lsid:zoobank.org:act:DE6F26BC-87E1-43B8-BDF9-47B25537627C.

Type species: *Parvucymoides dvorakorum* sp. nov.; by monotypy.

*Diagnosis:* As for the type species, as it is monotypic.

*Etymology*: The genus name is derived from a combination of the Latin words *parvus*, meaning little or tiny and *cymoides*, emphasizing the presumed systematic affinity of the species. The gender is male (masculine).

*Parvucymoides dvorakorum* sp. nov. ZooBank LSID: urn:lsid:zoobank.org:act:485FBA58-F578-48A0-AD3C-D93991C6A8D3.

*Type locality and age*: Trupelník hill near Kučlín u Bíliny (late Eocene)

*Etymology*: The species name is derived from the family name of the two brothers that collected the specimens (noun in the genitive case, gender: male (masculine), plural). Zdeněk Dvořák and Pavel Dvořák both collected numerous fossils in Kučlín since their childhood, and have largely contributed to the abundance of fossils available from this site.

*Species diagnosis*

Immature/male. *Body* elongate, bilaterally symmetrical. *Head* visible from dorsal view, roughly triangular in shape. *Compound eyes* visible in dorsal view (when preserved and accessible). *Antennula* with minimum of 12 articles; *antenna* with minimum of 10 articles, bases not in contact. *Anterior trunk* (pereon) *segment 1* narrowest, posterior margin evenly rounded, not encompassing the head. *Anterior trunk segment 7* wider than posterior trunk segment 1, posterolateral margins not overlapping lateral margins of posterior trunk segments. *Posterior trunk* (pleon) *segments* subequal in width, all narrower than pereon segments, posterior margins concave in dorsal view. *Pleotelson* narrower than pleon, wider than long. *Uropod* endopod and exopod sub-equal in length, extending past pleotelson posterior margin, apices narrowly rounded.

Female. Same as immature/male. *Body* longer and wider than males/immatures; *anterior trunk* (pereon) *segment 1* triangular, anterior margin encompassing the head.

*Remarks*

As the genus that has been created to accommodate this species is monotypic, this diagnosis contains a set of characters that distinguish the species from other extant and extinct species. This set of characters includes also those characters that could later serve as diagnostic characters of the genus or ‘genus diagnosis’, if a con-generic species to the herein presented species is described. This extensive diagnosis is referenced above according to ICZN Code Act 13.1.2.

*Material examined:*

*Holotype*. 1 male. P2339a/b as part and counterpart (8.44 mm TL; 4.04 mm W), collected at Kučlín, Czech Republic, during 1995–2010. Coll. Zdeněk Dvořák and Pavel Dvořák. Deposited at the National Museum, Prague, Figs. 2–3 (www.morphdbase.de/?S_VanderWal_20210812-M-154.1, www.morphdbase.de/?S_VanderWal_20210812-M-147.1).

*Paratypes*. 9 additional specimens. 2 males. P2346a/b part and counterpart (total body length & width not preserved), Figs. 6–7 (www.morphdbase.de/?S_VanderWal_20210812-M-153.1, www.morphdbase.de/?S_VanderWal_20210812-M-145.1). P2348 (7 mm TL, total width cannot be accurately determined), Fig. 8 (www.morphdbase.de/?S_VanderWal_20210812-M-152.1). 4 immatures? P2338a/b part and counterpart (7.41 mm TL, 2.95 mm W), Figs. 9–10 (www.morphdbase.de/?S_VanderWal_20210812-M-144.1, www.morphdbase.de/?S_VanderWal_20210812-M-151.1). P2347(at least 5.20 mm TL, 2.36 mm W), Fig. 11 (www.morphdbase.de/?S_VanderWal_20210812-M-143.1). P2344 (4.68 mm TL, 2.12 mm W), Fig. 12 (www.morphdbase.de/?S_VanderWal_20210812-M-149.1). P2343 (at least 6.12mm TL, at least 2.70 mm W), Fig. 13 (https://www.morphdbase.de/?S_VanderWal_20210812-M-142.1). 3 females? P2345a/b part and counterpart (at least 9.42 mm TL, 4.95 mm W), Figs. 14–16 (www.morphdbase.de/?S_VanderWal_20210812-M-150.1, www.morphdbase.de/?S_VanderWal_20210812-M-141.1,www.morphdbase.de/?S_VanderWal_20210812-M-148.1). P2341 (at least 9.39 mm TL, 6.20 mm W), Fig. 17 (www.morphdbase.de/?S_VanderWal_20210812-M-140.1). P2340 (at least 6.80 mm TL, total width cannot be accurately determined), Fig. 18 (www.morphdbase.de/?S_VanderWal_20210812-M-20.1). Same data as holotype.

*Additional material*. Male? P2342a/b part and counterpart (9.50 mm TL, total width cannot be accurately determined), Figs. 4–5 (www.morphdbase.de/?S_VanderWal_20210812-M-155.1, www.morphdbase.de/?S_VanderWal_20210812-M-146.1). Same data as holotype.

*Description of holotype* male (P2339a/b, Figs. 2–3)

One specimen as part (Fig. 2 with mostly dorsal features visible, P2339a) and counterpart (Fig. 3 with mostly ventral features visible, P2339b). Total body length 8.44 mm, total width 4.04 mm.

*Body* expanding in width posteriorly; longer than wide, 2.1x; widest at anterior trunk segment 5. *Head* triangular; wider than long, 1.5x; anterior margin narrowly rounded. *Eyes* not accessible.

Some articles of antennulae and antennae accessible. *Antennula* with at least nine articles; *antenna* with at least seven articles.

All *anterior trunk* (pereon) *segments* wider than long (Fig. 2), segment 1, 3.1x, not encompassing functional head; segment 2, 5.2x; segment 3, 6.2x; segment 4, 6.0x; segment 5 (widest), 5.7x; segment 6 (longest), 4.7x; segment 7 (posterior margin concave), 4.2x; all with at least one, partly preserved appendage.

*Anterior trunk appendages* (pereopods), distal region with 6 articles well accessible. *Proximal article* (coxa) accessible (Fig. 3C), as long as, or shorter than trunk segment.

*Trunk appendage 1* (thoracopod 2, right), basipod longer than wide, 1.9x; ischium longer than wide, 2.7x; merus twice as wide as long; carpus longer than wide, 1.7x; propodus wider than long, 2.2x; dactylus longer than wide, 2.1x.

*Trunk appendage 3* (thoracopod 4, right), basipod longer than wide, 2.2x; ischium longer than wide, 1.3x; merus as long as wide; carpus wider than long, 1.6x; propodus wider than long, 1.1x; dactylus twice as long as wide.

*Trunk appendage 3* (thoracopod 4, left), basipod longer than wide, 1.7x; ischium longer than wide, 1.7x; merus as long as wide; carpus wider than long, 1.4x; propodus wider than long, 1.6x; dactylus longer than wide, 2.7x.

*Trunk appendage 4* (thoracopod 5, right), basipod longer than wide, 1.5x; ischium wider than long, 2.4x; merus wider than long, 1.8x; carpus longer than wide, 1.5x; propodus longer than wide, 2.5x; dactylus twice as long as wide.

*Trunk appendage 6* (thoracopod 5, right), basipod twice as long as wide; ischium wider than long, 2.2x; merus wider than long, 1.2x; carpus wider than long, 1.1x; propodus longer than wide, 1.2x; dactylus longer than wide, 2.6x.

*Posterior trunk* (pleon) *segments* posterior margins concave (Fig. 2); all wider than long (Fig. 3), segment 1, 4.9x; segment 2 lateral margins not visible; segment 3, 7.0x; segment 4, 7.3x; segment 5 (longest), 4.5x; posterior trunk appendage insertion areas visible (Fig. 3).

*Pleotelson* (Fig. 3), converging to postero-medial point (possibly distorted); wider than long, 1.4x. *Uropods* with basipods extending past lateral margins of pleotelson; exo-and endopods distal margins not preserved/accessible, extending past pleotelson posterior margin.

*Variation*. The shape of the anterior margin of the functional head of specimen P2342 (Figs. 4 and 5) is broadly rounded. *Posterior trunk* (pleon) *segments* with lateral margins slightly extended. *Pleotelson* evenly rounded. Specimen P2346 (Figs. 6 and 7) have compound eyes visible, with at least six rows of ommatidia. Accessible antennula articles vary between at least five to six articles.

*Description of immature* (P2338a/b, Figs. 9–10)

One specimen as part (Fig. 9 with mostly dorsal features visible, P2338a) and counterpart (Fig. 10 with mostly ventral features visible, P2338b).

*Body* elongated; longer than wide, 2.5x; anterior trunk segments lateral margins sub-parallel.

*Head* sub-truncate oval; wider than long, 1.1x; anterior margin blunt, slightly rounded. *Eyes* not accessible.

Some elements of antennulae and antennae accessible (Fig. 10). *Antennula* with at least 12 articles; *antenna* with at least 10 articles.

All *anterior trunk* (pereon) *segments* wider than long (Fig. 9), segment 1, 3.5x, not encompassing functional head; segment 2, 4.8x; segment 3, 4.5x; segment 4, 4.3x; segment 5 (longest), 2.9x; segment 6, 4.0x; segment 7, 3.9x; all with at least one, partly preserved appendage (Fig. 10). *Trunk appendages* (pereopods), distal region with 6 articles well accessible. *Proximal article* (coxa) accessible (Fig. 10C), as long as, or shorter than trunk segment.

*Trunk appendage 1* (thoracopod 2, right) completely preserved without distortion, basipod longer than wide, 1.4x; ischium longer than wide, 1.2x; merus longer than wide, 1.2x; carpus longer than wide, 1.1x; propodus wider than long, 1.5x; dactylus longer than wide, 2.2x.

*Trunk appendage 1* (thoracopod 2, left) basipod longer than wide, 1.8x; ischium as long as wide; merus longer than wide, 1.1x; carpus as long as wide; propodus as long as wide; dactylus longer than wide, 3.1x.

*Posterior trunk* (pleon) segments with posteriorly angled, rounded, sub-parallel lateral margins; all wider than long, segment 1, 4.7x; segment 2, 4.5x; segment 3, 5.8x; segment 4 (shortest), 7.5x; segment 5, 5.1x; insertion areas of pleon appendages (pleopods) accessible (Fig. 10).

*Pleotelson* posteriorly evenly rounded; wider than long, 1.4x. *Uropods* endo-and exopod distal margins not clear, extending past pleotelson posterior margin.

*Variation*. The functional head of specimen P2347 (Fig. 11) is more sub-triangular than sub-truncate oval, with at least 6 antennulae articles accessible. Specimen P2344 (Fig. 12) have at least seven articles accessible. Specimen P2343 (Fig. 13) and specimen P2344 (Fig. 12) both have somewhat shorter posterior trunk segments with pleotelson shape varying between evenly rounded and sub-triangular. All pleotelsons are wider than long. The uropods of specimen P2343 (Fig. 13) extend only just past the pleotelson posterior margin.

*Description of female* (P2345a/b, Figs. 14–16)

One specimen as part (Fig. 14 with mostly dorsal features visible, P2345a) and counterpart (Figs. 15 and 16 with mostly ventral features visible, P2345b).

*Body* oval, longer than wide; widest at anterior trunk segment 4/5.

*Head* triangular; wider than long, 1.1x; with anterior margin narrowly rounded. *Eyes* not accessible.

Some articles of antennulae and antennae accessible. *Antennula* with at least six articles; *antenna* with at least four articles.

All *anterior trunk* (pereon) *segments* wider than long, segment 1 (longest), 2.4x, encompassing the functional head; segment 2, 3.3x; segment 3 (widest), 3.7x; segment 4, 5.1x; segment 5, 7.0x; segment 6, 5.7x; segment 7 left lateral margin not visible; segments 1–4 with at least one, partly preserved appendage.

*Trunk appendages* (pereopods) distal region with six articles well accessible. *Proximal article* (coxa) accessible (Fig. 16B).

*Trunk appendage 2* (thoracopod 3, right), basipod longer than wide, 1.7x; ischium wider than long, 1.2x; merus wider than long, 1.8x; carpus wider than long, 1.6x; propodus as long as wide; dactylus longer than wide, 3.3.

*Trunk appendage 3* (thoracopod 4, right), basipod longer than wide, 1.3x; ischium wider than long, 1.2x; merus wider than long, 3.7x; carpus wider than long, 3.0x; propodus as long as wide; dactylus longer than wide, 2.7x.

*Posterior trunk* (pleon) *segments* posterior margins slightly concave; segments 1, 2 & 5 lateral margins not visible; segments 1 and 2 lateral margins not visible; all segments wider than long, segment 3, 5.4x; segment 4, 7.9x; segment 5, 6.7x.

*Pleotelson*, *uropods* not preserved.

*Variation*. Specimen P2341 (Fig. 17) has the body widest at anterior trunk segment 4. The posterior trunk segments of specimen P2341 (Fig. 17) has slightly more extended lateral margins.

*Morphometric analyses*

**Figure 19:**
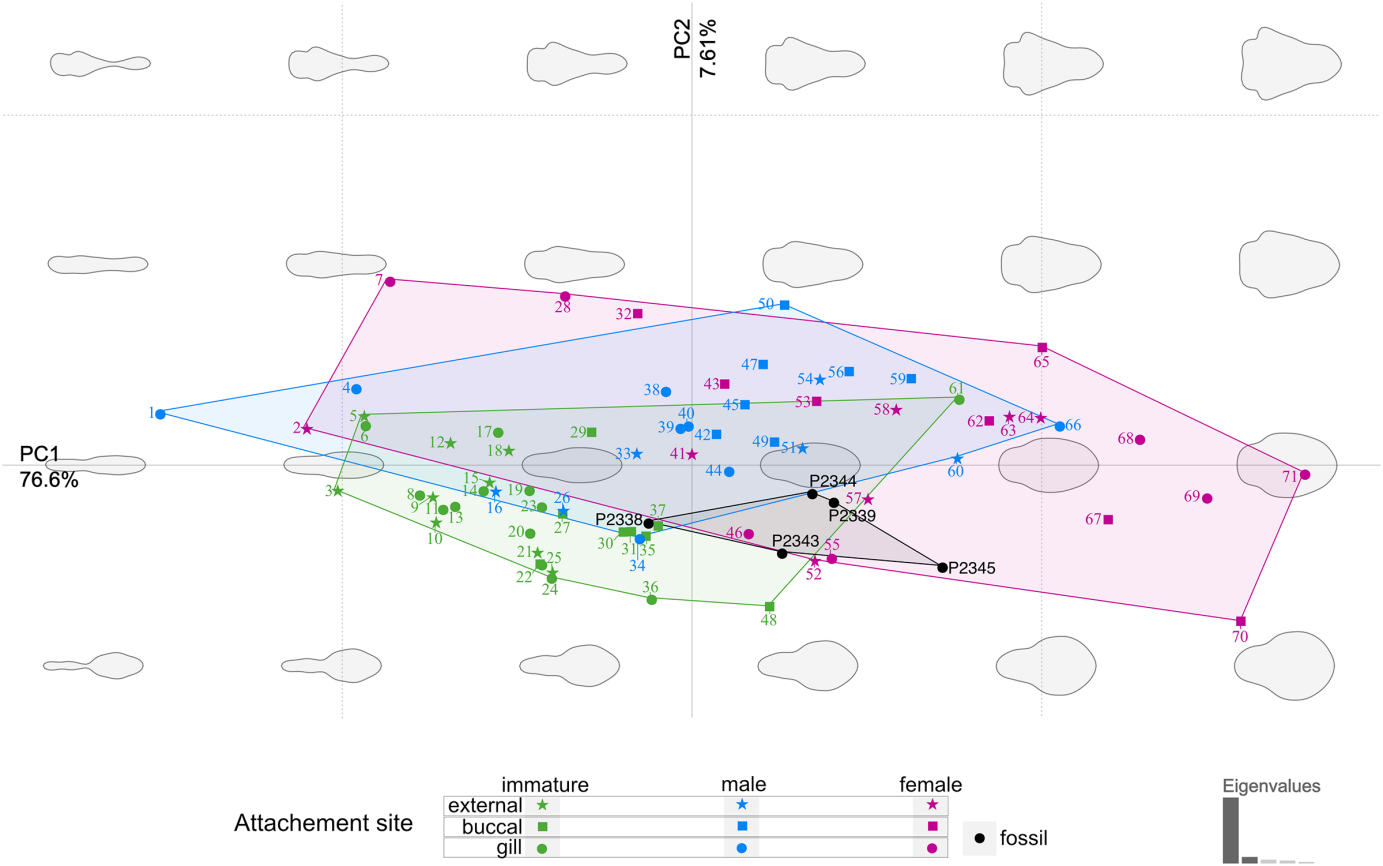
Principle component analysis representation of the body outline variation for all analysed specimens. Colour-coded according to their ontogenetic stage and shape-coded according to their attachment site. Numbers correspond to extant species included in the analysis: **1, 2, 10.** *Anilocra pilchardi* Bariche & Trilles, 2006. **3, 16, 57. ***Anilocra frontalis* Milne Edwards, 1840. **4,** **6, 7, 13.** *Olencira praegustator* (Latrobe, 1802). **5, 54, 58.** *Nerocila acuminata* Schioedte & Meinert, 1881. **8, 11, 19, 34, 55.** *Mothocya renardi* (Bleeker, 1857). **9, 15, 33, 41.** *Anilocra physodes* (Linnaeus, 1758). **12, 21, 26, 52.** *Anilocra pomacentri* Bruce, 1987. **14, 51, 64.** *Nerocila orbignyi* (Guérin-Méneville, 1832). **17**, 40, 68. *Agarna malayi* Tiwari, 1952. **18, 25, 60, 63.** *Nerocila bivittata* (Risso, 1816). **20, 24, 28, 38.** *Glossobius hemiramphi* Williams & Bunkley-Williams, 1985. **22, 47, 53.** *Ceratothoa gaudichaudii* (Milne Edwards, 1840). **23, 44, 71.** *Ryukyua circularis* (Pillai, 1954). **27, 32, 56. ***Ceraothoa* sp. **29, 30, 43, 50.** *Cymothoa liannae* Sartor & Pires, 1988. **31,** **41, 70.** *Cinusa tetrodontis* Schioedte & Meinert, 1884. **35, 45, 65. ***Cymothoa catarinensis* Thatcher et al., 2003. **36, 39, 46.** *Norileca indica* (Milne Edwards, 1840). **37, 42, 67.** *Ichthyoxenos puhi* (Bowman, 1962). **48,** **59, 62.** *Ceratothoa steindachneri* Koelbel, 1879. **61, 66, 69.** *Elthusa vulgaris* (Stimpson, 1857).

**Figure 20:**
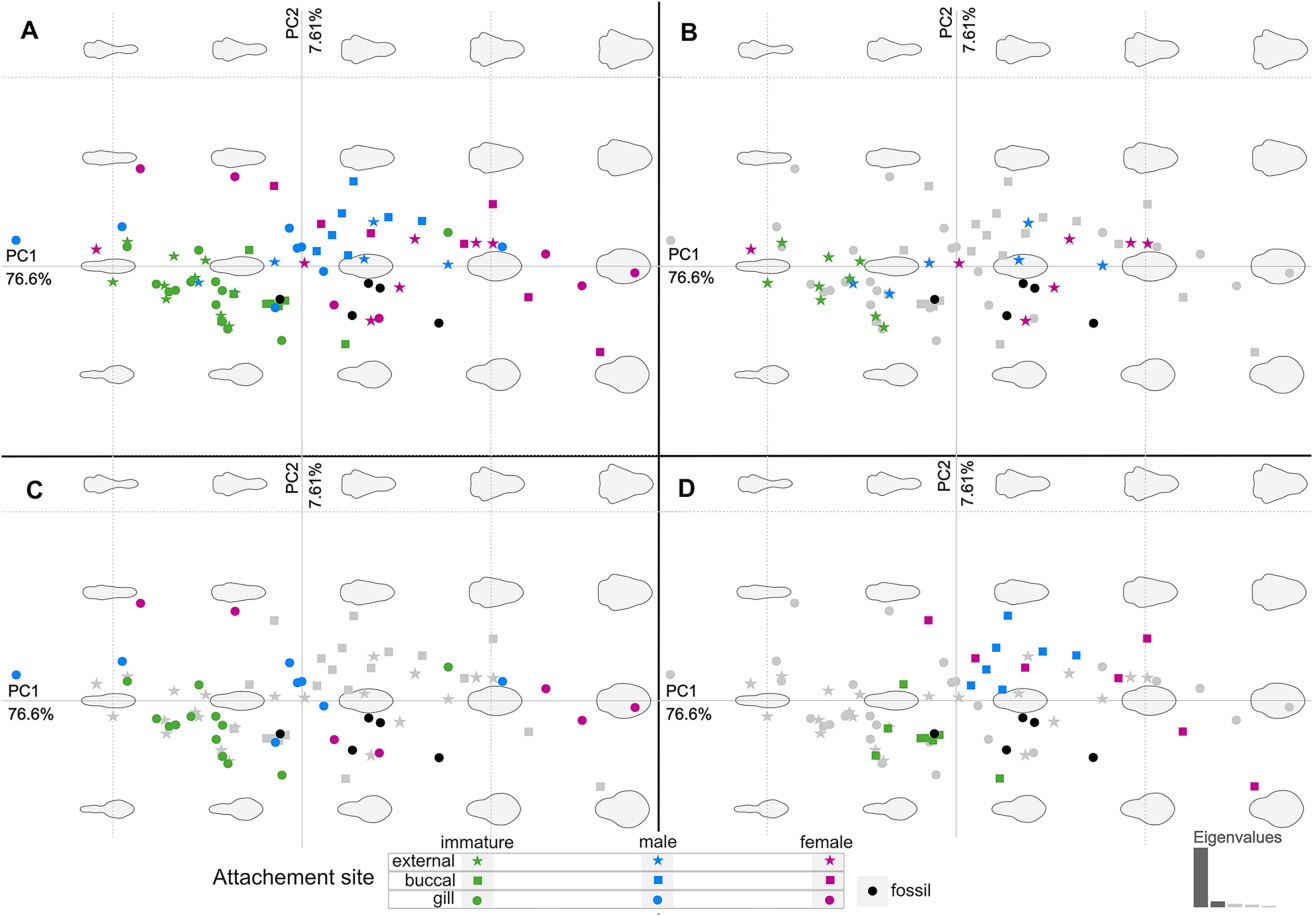
Principle component analysis representation of the body outline variation for all analysed specimens. Colour-coded according to their ontogenetic stage and shape-coded according to their attachment site. (A) Individuals of different ontogenetic stages and sites of attachment from extant species in colour. (B) Externally attaching individuals from extant species in colour. (C) Gill-attaching individuals from extant species in colour. (D) Buccal-attaching individuals from extant species in colour.

**Figure 21:**
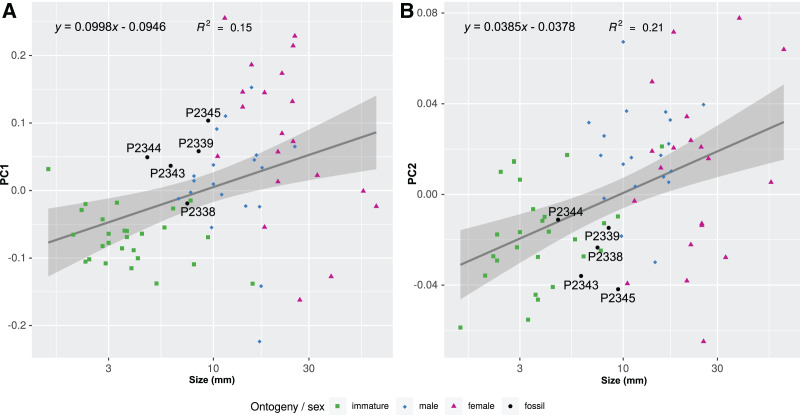
Shape parameters visualised in relation to the total body length (size, in mm) of each analysed specimen, extant and fossil. Linear models fitted to the first two principle components relative to the total body length. (A) PC1 to total body length. (B) PC2 to total body length.

The body outline variation for all analysed specimens, according to ontogenetic stage, is presented in Figs. 19 and 20. Only specimens P2338–P2339 and P2343–P2345 were reconstructed and used for the analyses, as these were preserved with complete length and width. These reconstructions are not perfect replications of the true shape of the specimens, but rather an idealised representation thereof, based on the interpretive drawings. For the presentation of results, only PC1 and PC2 were of interest, as they account for the most variation (see Fig. S1). PC1 and PC2 account for 84.2% of the total variation, with PC1 explaining 76.6% of the variation and PC2 explaining 7.6% of the variation. PC1 is largely influenced by the total body width, where the body is wider towards the positive values and narrower towards the negative values. PC2 is largely influenced by the region of the anterior trunk, where the body is most expanded in width. Positive values indicate a narrower anterior end and wider posterior end, while negative values indicate a narrower posterior end and wider anterior end. The general body shapes at specific PC values are visualised in the background of Figs. 19 and 20. The shape parameters are also visualised in relation to the total body length (size, in mm) of each analysed specimen. The relationship between PC1, PC2 and total body length is visualised in Fig. 21. Specimens from literature with no size data available, were excluded from the analysis (see Doc. S1).

## Discussion

The body segmentation and appendage pattern of *Parvucymoides dvorakorum* gen. et sp. nov. follows that of the group Eumalacostraca (6–8–6) (see Walossek, 1999). The uropods (specialised last trunk appendages) are apomorphic for Eumalacostraca (Walossek & Müller, 1998). There is no single apomorphic condition apparent in the examined fossils, which is not present in closely related groups. However, the following character states are indicative for Isopoda: body dorsoventrally flattened (Ax, 2000), and anterior trunk appendages without exopods (Ax, 2000; Wilson, 2009). The coxae are scale-like and fixed on the trunk (forming ‘coxal plates’) on trunk segments 2–7. This character state is apomorphic for Scutocoxifera (Dreyer & Wägele, 2002).

From representatives of *Urda*, the fossils of *P. dvorakorum* gen. et sp. nov. differ in having a much larger tergite of the anterior-most trunk segment (*e.g.*, Feldmann, Wieder & Rolfe, 1994). From representatives of Gnathiidae, the herein presented fossils differ in having seven pairs of well-developed appendages of the anterior trunk (see Boxshall & Montú, 1997; Smit & Davies, 2004). The examined fossils have well developed antennulae, unlike the shortened and modified antennulae of Epicaridea; uropods that are not styliform; and a morphology not reminiscent of epicaridium, microniscium, or cryptoniscium larvae (see Wägele, 1989; Brusca & Wilson, 1991; Schädel, Perrichot & Haug, 2019), therefore, excluding Epicaridea as having possible systematic affinity to the examined fossils.

Based on these systematically informative morphological characters, these specimens are interpreted as possible representatives of Cymothoidae, or at least closely related to Cymothoidae, during different developmental stages and are consequently interpreted as parasites.

**Figure 22:**
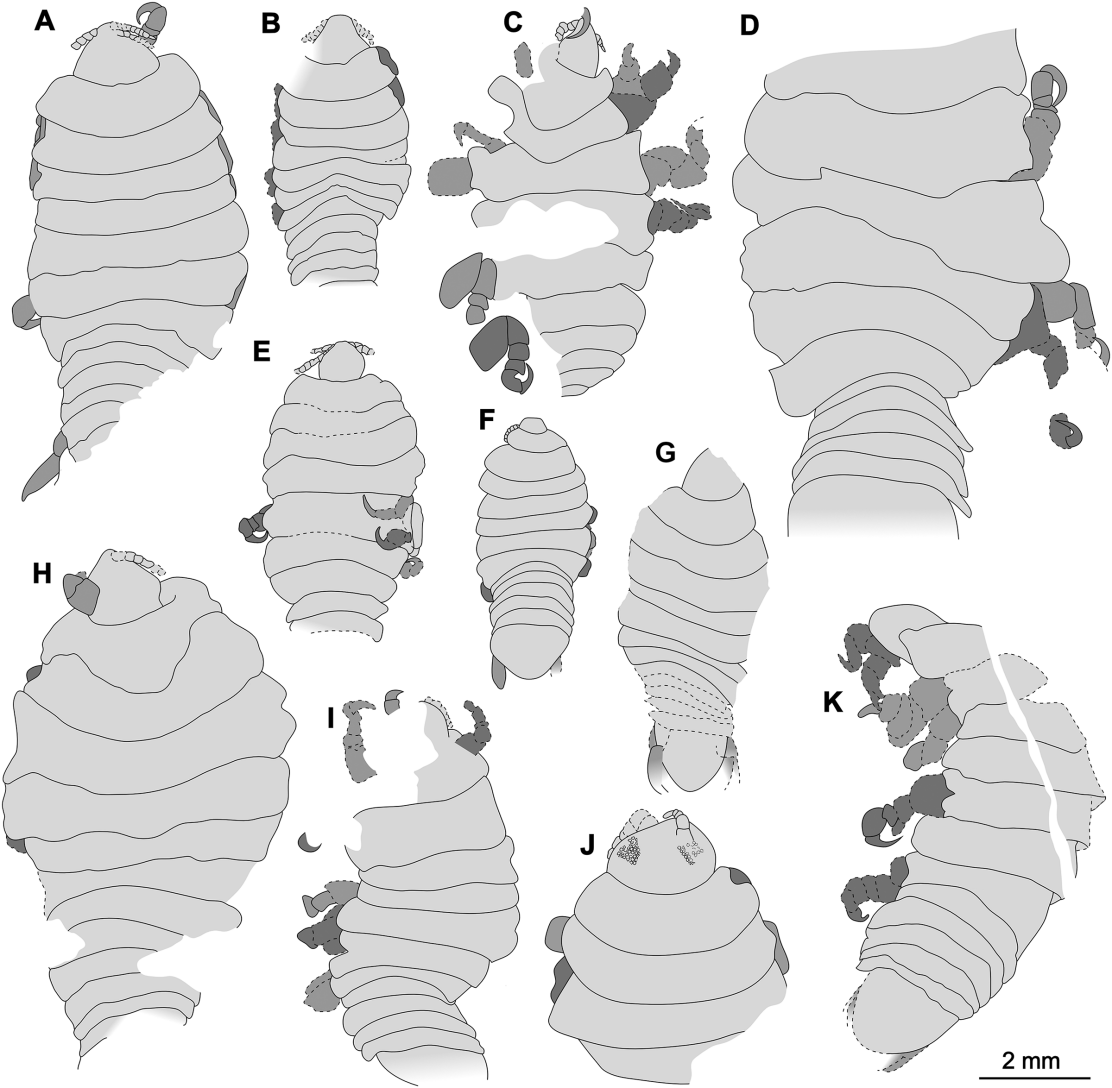
Comparison of the body size of examined fossils, same scale. (A) Specimen P2339. (B) Specimen P2347. (C) Specimen P2340. (D) Specimen P2341. (E) Specimen P2338. (F) Specimen P2344. (G) Specimen P2343. (H) Specimen P2345. (I) Specimen P2348. (J) Specimen P2346. (K) Specimen P2342.

Specimens examined herein range between a minimum length of 4.68 mm and a maximum of at least 9.50 mm, with larger, incompletely preserved specimens likely reaching a total body length of just slightly over 10 mm. The size comparison between the examined specimens is shown in Fig. 22. Mouthparts are not visible in the examined fossils. For the same reason, characters regarding setae can also not be assessed. In one specimen, P2346, compound eyes with clearly preserved ommatidia are preserved and located laterally on each side of the head. The eyes are not accessible from any of the remaining specimens. Similar to representatives of Cymothoidae, the examined specimens have anterior trunk appendages (thoracopods 2–8, pereopods 1–7) that each consist of seven articles and are prehensile, *i.e.*, specialised for attachment, with the distalmost article being a sharp, hook-like, curved dactylus (as seen from specimens P2338, P2339, P2340, P2341 and P2342). It is not possible to evaluate this aspect completely in the case of specimens P2345 and P2346, where only the anterior trunk appendages are preserved, and of specimen P2343, P2344, P2347 and P2348, where the trunk appendages are incompletely preserved or not visible. Even so, it is very likely that all herein studied specimens have 7 pairs of appendages with curved, hook-like dactyli, further inferring a parasitic life habit. In the specimens where they are preserved, the pleon segments 1–5 are free, with biramous uropods located antero-laterally on the pleotelson.

### Conspecificity

All herein studied type specimens are interpreted to be conspecific, as there are no apparent diagnostic characters that would suggest that they belong to separate species and all specimens were collected at the same location from within the same layers of rock. Some variation between specimens was noted to a similar degree in which extant conspecific individuals vary, and is therefore expected. Specimen P2342a/b seems to differ from the remainder of the specimens in the morphology of the pleon; however, this difference might be due to the mode of preservation (slightly distorted sclerites) rather than to a difference in the morphology of the once living animal. For this reason, this specimen is not included in the type series as a paratype, but rather as additional material examined.

### Morphological differences to other groups and species

The specimens examined herein share characters with many ingroups of Cymothoidae, but also lack, or vary from many diagnostic characters provided of extant groups, especially for different ontogenetic stages. *Parvucymoides dvorakorum* sp. nov. can be distinguished from extant species of Cymothoidae by: its small overall body length, especially immature and male stages, not exceeding much more than 10.0 mm, adult female specimens might be somewhat larger; an ovoid, but symmetrical body shape of larger (adult) specimens; having 12 or more antennulae articles.

Only a few extant species of Cymothoidae have a comparable, small body length as adult females, such as: *Artystone minima* Thatcher & Carvalho, 1988 (5.2–6.9 mm); *Catoessa ambassae* Bruce, 1990 (7.5–9. 3 mm); *Joryma brachysoma* (Pillai, 1964) (10.5–13.6 mm, Aneesh, Helna & Kumar, 2019); *Elthusa samariscii* (Shiino, 1951) (10–13.4 mm, Kumar & Bruce, 1997, Aneesh et al., 2020 and *Elthusa sigani* Bruce, 1990 (9.5–13.0 mm)); *Mothocya argenosa* Bruce, 1986 (5.5–9.8 mm); *Mothocya bertlucy* Hadfield, Sikkel & Smit, 2014 (7.0–9.0 mm); *Mothocya epimerica* Costa, 1851 (5.5–11.5 mm, Bruce, 1986); *Mothocya powelli* Van der Wal et al., 2021 (7 mm), *Mothocya waminda* Bruce, 1986 (5.6–8.9 mm); *Mothocya bermudensis* Bruce, 1986 (8.8–9.8 mm); *Mothocya rosea* Bruce, 1986 (6.2–8.4 mm); *Nerocila lomatia* Bruce, 1987 (7.0 mm (male)–16.0 mm); *Norileca triangulata* (Richardson, 1910) (9.2–18 mm, Rameshkumar & Ravichandran, 2015, Bruce, 1990); *Telotha henselli* (von Martens, 1869) (6.0–14 mm, Taberner, Volonterio & De León, 2003).

*Parvucymoides dvorakorum* gen. et sp. nov. can be distinguished from the genera of the above mentioned, similar-sized species. The ovoid and laterally symmetrical body shape of *P. dvorakorum* gen. et sp. nov. distinguishes it from the asymmetrical or strongly twisted body shapes of female individuals of *Joryma* Bowman & Tareen, 1983 (see Aneesh et al., 2019.), *Norileca* Bruce, 1990 (see original description) and *Mothocya* Costa in Hope, 1851 (see Bruce, 1986; Aneesh et al., 2016). The subtriangular to truncate functional head distinguishes *P. dvorakorum* gen et sp. nov. from *Nerocila* Leach, 1818 (see Bruce, 1987a; Nagler & Haug, 2016) and *Telotha*.

Schioedte & Meinert, 1884 (see original description and Taberner, Volonterio & De León, 2003) as representatives of the latter two groups both have a broadly rounded functional head anterior margin. A closer relationship to *Nerocila* can immediately be excluded, based on numerous characters including: larger size; pleon morphology; and slender uropod exopods which are longer than the endopods.

*Telotha* and *Artystone* Schioedte, 1866 (see Thatcher & Carvalho, 1988; Thatcher & Schindler, 1999) both have antennulae and antennae with between eight to nine articles, compared to the 10–12 minimum of the genus described here. The antennulae in species of *Catoessa* Schioedte & Meinert, 1884 (see Bruce, 1990) and *Mothocya* are thicker (‘more stout’) than the antennae, where these are subequal in thickness in *P. dvorakorum* gen. et sp. nov. Regarding anterior trunk segments, *Joryma* can be excluded based on the largely produced anterolateral margins of anterior trunk segment 1 in the adult females, as well as the anterior trunk segment 7 that overlaps posterior trunk segment 1 lateral margins. The latter character difference is also noticeable in *Mothocya* and *Elthusa* Schioedte & Meinert, 1884 (see Bruce, 1990; Kumar & Bruce, 1997). The coxae in the examined fossils are not well accessible and visible in all specimens, but from what is accessible, these differ from the large, rounded coxae of *Mothocya* and the posteriorly produced, acute coxae in *Nerocila*; in both groups extending to, or past the corresponding trunk segment posterior margin.

The trunk appendages of the examined fossils of *P. dvorakorum* gen. et sp. nov. all have long, acute dactyli, in contrast to the trunk appendage 7 of *Artystone*, of which the dactylus is short (less than half the length of the propodus) and distally round. Considering posterior trunk segments (pleon), those of *Catoessa* and *Elthusa* are notably different. Species of *Elthusa* have a wide pleon (mostly equal in width or wider than anterior trunk segment 7); while representatives of *Catoessa* have laterally extended pleon segments, with gaps between the segments. Representatives of the group *Catoessa* additionally have a unique, rotationally twisted posterior trunk. Posterior trunk (pleon) segments (pleonites) of *P. dvorakorum* gen. et sp. nov. are narrow with no gaps. Many of these extant groups have notable differences in pleotelson morphology. The posterior margins of the pleotelson of *P. dvorakorum* gen. et sp. nov. are subtriangular to broadly rounded in all specimens where it is accessible; slightly and wider than long. Representatives of *Joryma* (males), *Telotha* (immatures and males) and *Artystone* have a pleotelson that is longer than wide, with that of *Telotha* converging to a posteromedial point (in immatures and males) and that of representatives of *Artystone* being subtriangular to heart shaped. Lastly, the shape of uropods provides clear distinctions. *Parvucymoides dvorakorum* gen. et sp. nov. has uropods with the endopod and exopod subequal in length, longer than uropod basipod, extending slightly past pleotelson posterior margin. Representatives of both *Mothocya* and *Artystone* also have the exopods longer than the endopods, with representatives of *Artystone* additionally having uropod basipods longer or as long as the rami.

From the results of the body shape analysis (Figs. 19 and 20), it is clear that most of the body shape data points of the examined fossils are in close to very close proximity of those of various developmental stages of extant species. The body shapes of the *P. dvorakorum* sp. nov. specimens included in the analysis, can further be compared to various extant species with similar body shapes in order to further substantiate its interpretation as a separate species.

The extant species and their representative ontogenetic stages that have the most similar body shapes (according to Figs. 19 and 20) to the examined fossil specimens are: the externally attaching *Anilocra frontalis* Milne Edwards, 1840 (female), *Anilocra pomacentri* Bruce, 1987 (female), and *Nerocila orbignyi* (Guérin-Méneville, 1832) (male); the gill attaching *Mothocya renardi* (Bleeker, 1857) (male, female) and *Norileca indica* (Milne Edwards, 1840) (female, twisted body shape straightened); and the buccal attaching immature stage 2 (manca) of *Cinusa tetrodontis* Schioedte & Meinert, 1884; *Cymothoa catarinensis* Thatcher et al., 2003; *Cymothoa liannae* Sartor & Pires, 1988; *Ichthyoxenos puhi* (Bowman, 1960).

*Norileca*, *Nerocila* and *Mothocya* have already been excluded as possible affinities for the examined specimens of *P. dvorakorum* gen. et sp. nov. (above). Specimens P2339 and P2343 plot within close proximity of two species of *Anilocra* Leach, 1818 (females) (Figs. 19 and 20), which can be differentiated by having a larger overall body size; a pleotelson that is longer than wide; trunk appendage 7 notably longer than trunk appendage 6; and with antennulae usually with eight articles.

Specimen P2338, interpreted as immature (stage 3, juvenile), has a body shape similar to the immatures of *C. tetrodontis, C. catarinensis*, *C. liannae* and *I. puhi* and to the male stage of *M. renardi*. During immature stage 2, the anterior trunk segment 7 is underdeveloped and with underdeveloped trunk appendages. The illustration and descriptive characters available for immatures of *C. tetrodontis* do not allow for a sufficient comparison between this ontogenetic stage and specimen P2338. Even so, the later developmental stages of *C. tetrodontis* can be compared to and distinguished from *P. dvorakorum* sp. nov. by having the proximal articles of the antennae close together, almost in contact; a short anterior trunk segment 1; posterior trunk segment 1 (pleon segment 1) notably narrower than the remaining pleon segments; and uropods that do not reach the posterior margin of the pleotelson. The immature stage 2 of *C. catarinensis* can be distinguished from specimen P2338 by having fewer antennulae and antennae articles (eight, *vs*. 10–12 minimum) and uropods that extend well past the pleotelson posterior margin. The body shapes of adult stages of *C. catarinensis* (male and female) do not compare to those of any of the examined specimens. Even though specimen P2338 plots close to the immature stage 2 of *C. liannae*, its body shape outline is not similar to that of the immature stage 3 (juvenile) or adult stages of the latter species. The immature stage 2 of *C. liannae* has uniquely long antennae, reaching to anterior trunk segment 6. These antennae are much shorter during all later developmental stages. It further has uropod rami that extend far beyond the pleotelson posterior margin. The immature stage 2 of *Ichthyoxenus puhi* can be differentiated from specimen P2338 by having a larger, broadly rounded functional head and shorter, wider, broadly rounded uropod rami that don’t extend to the pleotelson posterior margin. Specimen P2338 is in close proximity of the male representative of *M. renardi*, but not of the immature stages 1–2. *Mothocya renardi* male stages have narrower and longer uropod rami that extend well beyond the pleotelson posterior margin and pleon segments wider than anterior trunk segment 7. Therefore, the examined fossils cannot be interpreted as representatives of these species.

### Ontogenetic interpretation

**Figure 23:**
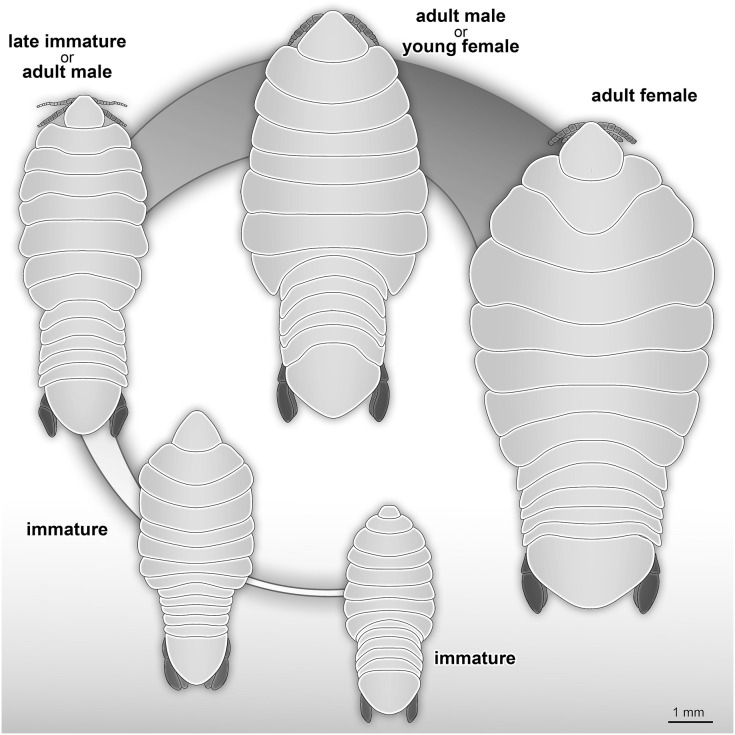
Reconstruction drawings of interpreted ontogenetic stages of the examined fossil specimens. (A) Specimen P2344 (immature). (B) Specimen P2343 (immature). (C) Specimen P2338 (immature/young male). (D) Specimen P2339 (adult male/young female). (E) Specimen P2345 (adult female).

The life cycle and developmental stages of representatives of Cymothoidae are consistent (see Smit, Bruce & Hadfield, 2014), and have been described and illustrated for various extant groups, for example *Anilocra* Leach, 1818; *Agarna* Schioedte & Meinert, 1884; *Ceratothoa* Dana, 1852; *Glossobius* Schioedte & Meinert, 1883; *Mothocya* Costa in Hope, 1851; *Nerocila* Leach, 1818; and *Norileca* Bruce, 1990 (see Brusca, 1978; Adlard & Lester, 1995; Mladineo, 2003; Bakenhaster, McBride & Price, 2006; Aneesh et al., 2016, 2018; Kottarathil et al., 2019). Species of Cymothoidae are protandrous hermaphrodites, where males develop and moult into adult females under certain conditions (Legrand, 1952; Trilles, 1991; Bunkley-Williams & Williams, 1998). This change in sex during ontogeny differentiates adult male and adult female specimens as two different ontogenetic stages. This sexual dimorphism, that also affects the general shape of the body, is well documented for Cymothoidae in terms of primary sexual characters and apart from appendage dimorphism (Bunkley-Williams & Williams, 1998; Bruce, 2002; Poore & Bruce, 2012). Thus, adult male and female specimens can be well differentiated. More recently, detailed morphological descriptions and differentiating characters of different immature stages have been presented (Bakenhaster, 2004; Jones et al., 2008; Aneesh et al., 2016; Van der Wal & Haug, 2020). A tentative restoration of the ontogenetic sequence of the examined fossils (Fig. 23) appears very similar to that in modern day representatives of Cymothoidae.

### Possible immature representatives

The term ‘immature’ is used here to refer to all stages after hatching (post-marsupial development), but before maturation (*sensu* Van der Wal & Haug, 2020). Immatures of extant species have a larger body length to width ratio (more elongated) that decreases over ontogenetic development (Fig. S2). This results in adults that have a smaller length to width ratio (more rounded) (for example, see figures and illustrations from Trilles, Colorni & Golani, 1999, fig. 4; Thatcher, de Lima & Chellappa, 2007, figs. 23, 46; Aneesh et al., 2019, fig. 1; Van der Wal & Haug, 2020, figs. 1, 4, 7,11, 14, 17, 20, 26, 29). Even though the body shape is highly variable among extant representatives of these groups, there seems to be a trend throughout, that adults are less elongated than immatures of the same species (see interpretation: attachment site). The source of this variation is seen at the mid-to posterior region of the anterior trunk, including the anterior region of the posterior trunk. The anterior and posterior ends of the specimens show variation to a much lesser extent (Fig. S2). Immature and adult male specimens of Cymothoidae have not been as thoroughly documented (described, photographed or illustrated) as adult female specimens, even though changes in body shape and size are prominent through these developmental stages.

The examined fossil specimens P2338, P2343, P2344 and P2347 have the same type of slender and elongated body, most prominent in specimen P2338 (Figs. 9 and 10), as in many immature stages of extant species. The body ratio trend is also noted with the specimens studied here. Specimens P2338, P2343, P2344 and P2347 have a body length range of 4.68–7.41 mm and a width range of 2.12–2.95 mm, resulting in an average body length to width ratio of 2.38. This ratio is notably higher than the body ratio of the specimens herein interpreted as adult representatives (see discussion ‘Possible adult representatives’).

Considering the ordinated (PCA) values of the body shapes (Figs. 19 and 20), the reconstructed body shapes of specimens P2338, P2343 and P2344 fall well within the shape variation of immatures, with specimen P2338 notably close to extant immature representatives *Cinusa tetrodontis* Schioedte & Meinert, 1884; *Cymothoa catarinensis* Thatcher et al., 2003; *Cymothoa liannae* Sartor & Pires, 1988; *Ichthyoxenos puhi* (Bowman, 1960); and a gill attaching male of *Mothocya renardi* (Bleeker, 1857). The body shape of specimen P2343 is similar to some extant female representatives: *Anilocra pomacentri* Bruce, 1987 (Bruce, 1987b, external attaching) and *Norileca indica* (Milne Edwards, 1840) (gill attaching), requiring further consideration regarding the substantiation of the interpreted ontogenetic stage. The same is true for the shape of specimen P2344, which is similar in thickness to those of extant males (*Ryukyua circularis* (Pillai, 1954) and *Nerocila orbignyi* (Guérin-Méneville, 1832)) and a female (*Anilocra frontalis* Milne Edwards, 1840). The body shape most similar to this is that of specimen P2339 (herein interpreted as a male), which is only slightly wider (relative to body size) than specimen P2344. In order to further substantiate the ontogenetic interpretation of specimens P2343 and P2344, the total body length (size) of all analysed specimens is considered.

With regards to size, the body measurements for specimens P2338, P2343, P2344 and P2347 are the smallest of the examined specimens. With regards to body width, the reconstructed body shapes of specimen P2343 and P2344 are relatively wider than most example immatures analysed (Figs. 19 and 20), but with the area of greatest width (widest in the medial region of the body, PC2), similar to those of extant immatures. The results from both body shape and size analyses support the interpretation of specimen P2338 as an immature individual. The interpretation of specimen P2343 and P2344 as immatures is supported by the size comparison and region of greatest body width (PC2), but partially supported by the total body width analysis (PC1). The interpretation of specimen P2347 as immature is based on general body shape and size comparison among the examined specimens.

Immature forms of Cymothoidae have different developmental stages (*e.g.*, pre-mancae, mancae and juveniles/natatory-stage individuals, *sensu* Van der Wal & Haug, 2020). These can be differentiated based on characters such as the presence or absence of developed appendages on trunk segment 7, the presence of yolk and the presence of setae on the pleopods and uropods. Since the latter two characters are not visible in the examined fossils, due to the mode of preservation, the exceptionally preserved trunk appendages allowed for a more accurate interpretation.

Specimens P2338, P2343, P2344 and P2347 are interpreted as representing the final immature stage (immature stage 3 *sensu* Van der Wal & Haug, 2020; ‘juvenile’ *sensu* Brusca, 1978; Segal, 1987; Kottarathil et al., 2019; ‘natatory-stage’ *sensu* Jones et al., 2008) for the following reasons. Immature stages prior to immature stage 3 (*i.e.*, immature stage 1 and 2, also referred to as pre-manca and manca stage respectively) lack fully developed appendages on the posterior-most segment of the anterior trunk (thoracopod 8, pereopod 7). The appendages on this segment are fully developed at immature stage 3 (Aneesh et al., 2018; Boyko & Wolff, 2014; Sartor & Pires, 1988; Jones et al., 2008). These seven pairs of well-developed trunk appendages are best visible from specimen P2338 as immature (Fig. 10).

### Possible adult representatives

Examined specimens P2339–P2342, P2345–P2346 and P2348 are interpreted as at least immature adults (immature males or immature females). Since neither adult male characters (*e.g.*, appendix masculina on pleon appendage 2 and penes), nor adult female characters (*e.g.*, developed brood pouch, no penes) are visible on the fossils, this interpretation is based on the body shape and size.

When considering the overall body shape and individual size of these specimens (Figs. 19 and 20), a further differentiation between possible male and female specimens can be made. Specimens P2339, P2342, P2346, P2348 are herein interpreted as possible male or transitional stage individuals, while specimens P2340, P2341 and P2345 are interpreted as possible female specimens. The body shape variation that suggests this distinction, is most prominent from comparing the reconstructed body shapes of specimen P2339 and specimen P2345. The remaining fossil specimens were either incompletely preserved or preserved at an angle so that no reconstruction could be done. Therefore, the interpretation of the remaining specimens is based on general body shape and size comparison.

Specimen P2339 has a slightly less elongated, pear-like body shape, widening towards the posterior end, widest at trunk segments 5 or 6 (Fig. 2). Preserved with minimal dorsal and ventral feature distortion, this specimen has a similar body shape to that of extant male representatives of Cymothoidae. When considering the results of the shape analysis (Fig. 19), the reconstructed body shape of the supposed male specimens group between data points of male and female representatives. This indicates that specimen P2339 has a body shape comparative to males or small females of extant, externally attaching species (*Anilocra frontalis* and *Nerocila orbignyi*), suggesting a possible transitional stage from male to female. With regards to the body width, specimen P2339 is comparable to most herein analysed males (Fig. 21A) with the area of greatest width (Fig. 21B) still within the range of extant males (Figs. 19–21). The results from both body shape and size analyses support the interpretation of specimen P2339 as a transitional stage specimen, between the stages of adult male and becoming an adult female.

The possible male/transitional stage individuals (specimens P2339, P2342, P2346 and P2348) have a body length range of 7.0–9.5 mm and a width of 4.0 mm (with only the width of specimen P2339 available). This results in an average body length to width ratio of 2.04, corresponding to the trend of a smaller ratio of adult male specimens compared to that of immatures.

Possible female specimens P2340, P2341and P2345 have body proportions that are somewhat different to those interpreted as male representatives. These specimens have an oval to rounded anterior trunk region, with the body widest at trunk segment 3 or 4. This oval body shape is especially prominent within adult female stages of many extant species, suggesting that these specimens might be female representatives. With a body length range of >6.80–>9.42 mm and a width range of 4.95–6.20 mm, these specimens are the largest among the examined fossils, when incomplete length preservation are taken into consideration. These measurements result in an average body length to width ratio of 1.46, which is smaller than that of the fossil specimens interpreted as adult males and immatures.

According to the body shape analysis results from Fig. 19, the possible female reconstructed body shape (P2345) plot within the group of female representatives of extant species, surrounded only by other adult female body shapes (*A. pomacentri*, *A. frontalis*, external attaching; *M. renardi*, gill attaching). Even though its overall body size is smaller than that of the analysed extant females, specimen P2345 has a similar relative body width to extant species (Fig. 21A), but with the area of greatest width more toward the anterior region (Fig 21B) than most extant females included in the analysis.

In addition to these characters, another female specific feature was noted: specimens interpreted as possible females have a rather distinct trunk segment 1 shape. This structure is almost triangular in shape, with the posterior margin medially elongated; and encompassing the head from the lateral sides (as seen in specimens P2340 and P2345, Figs. 18, 14). Specimens interpreted as possible immatures, males and transitional stages, have a trunk segment 1 with narrowly rounded antero-lateral angles and with an evenly rounded posterior margin (as seen in specimens P2339, P2342, P2343 and P2346, Figs. 2, 4, 13, 6). This structure is incompletely preserved in specimens P2341 and P2348.

### Body shape as a proxy for ontogenetic stage

The comparison of body shapes (Fig. 19) show no distinct separation between ontogenetic stages among various extant species of Cymothoidae. These results may be different for an intra-species analysis. Even so, when body shape is compared relative to actual size (Fig. 21), a general but weak trend becomes visible. These trends were noticed for the individuals included in the analysis from literature: 18 extant species, with representatives that attach to different sites on the host (mouth, gills, external). A larger dataset would be needed to further support these trends:

(1) Immature individuals of extant species tend to have a smaller range in body width, generally having slender/narrow body outlines (grouping mostly within the negative PC1 values in Figs. 19–21). This narrow body shape is characteristic of most externally attaching forms, independent of their ontogenetic stage. The immature specimens included here, range in size between 1–10 mm, with only one individual (*Nerocila acuminata* Schioedte & Meinert, 1881) grouping outside of this size range, with an average body length of 15.7 mm, as calculated from Segal (1987). The latter individual also plots in close proximity (shape and size) to two other male representatives of *Anilocra*.

(2) Body shapes tend to become more diverse through development, with adult males having a larger range in body width than immatures. Their body size range is between 7.7–17.5 mm, with only the male representative of *Ceratothoa* reaching a size of 25.5 mm. This is not surprising, since species of *Ceratothoa* are some of the largest in size, if not the largest, among the ingroups of Cymothoidae.

(3) Female individuals are highly diverse in body shape and size, even more so than male representatives, supporting the notion of wide morphological variability among ingroups of Cymothoidae. In species of Cymothoidae, the body size ranges between 10.5–65.0 mm and the body shape ranges (in body width, PC1) from long, slender individuals, as seen with *Anilocra pilchardi*, to strongly oval to round individuals, as seen with *R. circularis and C. tetrodontis*.

Not surprisingly, adult females occupy the largest area in our ordinations, indicating that this ontogenetic stage is the most morphologically variable. This can be explained by the ecology and life habit of adult females of Cymothoidae as permanent parasites of mainly fish hosts. The site of attachment to the host plays a distinct role in the final body shape of female individuals due the space available for growth (Kensley, 1978; Brusca, 1981; Hadfield, 2012).

### Possible site of attachment

The body shape outline analysis of the included extant species can provide insight into the possible site of attachment of *P. dvorakorum* sp. nov. (Fig. 20). Even though there is no obvious trend, it is noticeable that all immatures have long, slender bodies (with only two exceptions: *E. vulgaris* (Stimpson, 1857) and *C. steindachneri* Koelbel, 1879) and how, throughout development, species that attach to different sites develop differently shaped, wider bodies. According to the results (Fig. 20), externally attaching species have the most constant length to width ratio and only slightly gain some width through development along the midline of the body (with *A. pilchardi* Bariche & Trilles, 2006 as exception). Even though attaching to the external surface of a host does not pose any growth restrictions, it causes the resulting adult body shape to be streamlined, in order to withstand the water current and flow. Gill-attaching species have more variation in midline width, according to the available space in the gill cavity of the host. Gill-attaching species usually have rounded and strongly twisted body shapes in order to take on the shape of the space available in the gill cavity. Buccal-attaching species do not show as much variation in width, but the most variation in where the increase in body width takes place (*i.e.*, towards the anterior part of anterior trunk or toward the posterior part of anterior trunk). The growth in width of buccal-attaching species are restricted in the mouth cavity of the fish, resulting in elongated slender (almost cylindrical) adults, that gain body width depending on available space.

The position of the examined fossil specimens in Fig. 20 does not clearly suggests a possible site of attachment. It does, however, show that especially the fossils interpreted as adult male and female are less likely to have been buccal-attaching, as the body shapes of buccal-attaching species are the least similar to the reconstructed fossil body shapes. Extant male and female individuals of externally-attaching groups seem to have the most similar body shapes to the interpreted male and female specimens examined here. The isolated finds of these specimens support the possibilities that they might have been either buccal-attaching or externally-attaching, based on the ability of extant buccal- and externally-attaching species to abandon their host when it is dying. Gill-attaching species cannot easily detach from the host and leave the gill cavity, therefore, dying *in situ*. Although not conclusive, it is most likely that the examined specimens were externally-attaching individuals, based on this ecological strategy and the results presented in Fig. 20.

### Palaeoecology

All examined fossil specimens are isolated, showing no interaction or closeness to other macro-organisms. Immatures of Cymothoidae are free-swimming, in search of an appropriate fish host to attach to. This might explain why the immature specimens are preserved isolated from potential hosts. The lack of a fish host in close proximity to the fossils does not exclude the possibility that the studied specimens were permanent parasites, but is likely a result of their ontogenetic stage as immature individuals. Adult representatives are usually permanently attached to a host. Yet, the specimens interpreted here as possible adult representatives are also isolated. Even though it is unlikely for adult specimens of Cymothoidae to be encountered without a host, it is not impossible. The death of a host could result in the parasitic individual detaching from it, in order to find a new host. Alternatively, the individuals might have accidentally become detached from its host. If the studied fossils were permanent parasites, isolated discoveries are certainly not unlikely.

To date, no possible specimen of Cymothoidae has been discovered attached to a fish fossil at this collection site. Preserved fish bones are small, and if there was to be a parasite preserved in the mouth or gill areas of a fish, in most cases it would be hard to recognise.

By considering the reconstructed palaeoenvironment from which the fossils were collected, it is possible to speculate on the life habit of the studied individuals, based on the ecology of extant animals (actualism). The presence of temperate basses (Moronidae, ray-finned fish) in the depositional environment indicates a possible connection to the sea *via* rivers (Micklich, 1990; Micklich & Böhme, 1997; Přikryl, 2008) and additionally points out possible hosts for the studied individuals. Today, temperate basses occur in marine, fresh- and brackish water habitats (Wallace, 1971; Whittier, Halliwell & Daniels, 1999; Jobling, Peruzzi & Woods, 2010), with some records of species infested with species of Cymothoidae (Sadzikowski & Wallace, 1974; Papapanagiotou, Trilles & Photis, 1999; Charfi-Cheikhrouha et al., 2000; Bariche & Trilles, 2006; Hata et al., 2017). If Cymothoidae-like parasites were associated with representatives of Moronidae from this collection site, such findings are expected to be rare, as there are only two representatives of Moronidae fossils recorded, where the mouth and/or pharyngeal region of the fish is preserved.

### Records and origin of freshwater parasitic isopods

The sediments from which the fossils were collected were most probably deposited in a freshwater lake (see Geological setting and palaeoenvironment). This suggests that the fossil specimens collected from these sediments were freshwater inhabitants. Even though a large majority of extant species of Cymothoida are distributed in marine environments, many ingroups, including Cymothoidae, have been recorded from freshwater and brackish water habitats (Smit, Bruce & Hadfield, 2014; Tavares-Dias et al., 2014; Hata et al., 2017).

There is no concise distribution pattern for representatives of Cymothoidae in freshwater. Yet, the majority of cases have been reported from South American freshwater sources (Huizinga, 1972; Bowman, 1986; Bastos & Thatcher, 1997; Lins et al., 2008; Tavares-Dias et al., 2014), with some species recorded from central African (see Moore, 1898; Van Name, 1920; Fryer, 1965, 1968; Lincoln, 1971) and Asian freshwater environments (Tsai & Dai, 1999; Yamano, Yamauchi & Hosoya, 2011). Some species have been reported from estuaries in North America (Lindsay & Moran, 1976) with one record from southern Europe (Mediterranean) (see Leonardos & Trilles, 2004).

The occurrence of the examined fossil specimens in sediments from a fossil freshwater lake not only suggests the presence of freshwater forms of Cymothoidae in Europe, it also suggests that the transition between the marine and the freshwater lifestyle happened during or even before the Eocene. The co-occurrence of temperate basses (Moronidae) as possible fish hosts provides a possible scenario how this transition might have happened: through the colonisation of freshwater habitats by fishes from the ocean. Alternatively, the fossil specimens could represent remains of individuals that were transported to the lake by anadromous migrating fish.

## Conclusions

The examined fossils are conspecific and interpreted as ingroup representatives of, or close relatives to, the group Cymothoidae. Fossils of the newly described species, *Parvucymoides dvorakorum* gen. et sp. nov., possibly represent different developmental stages. The examined fossil specimens (and subsequently the new species) have been interpreted as parasites based on their close affinity to Cymothoidae as well as the presence of seven pairs of thoracopods with prehensile, curved and hook-like dactyli. Ray-finned fishes occurring in the same palaeoenvironment might possibly have been the hosts of these parasites. The interpretation of the ontogenetic stage of the fossils is based on an analysis of the body sizes and different morphological characters of extant representatives of Cymothoidae and the fossils. The palaeoenvironment suggests that these individuals once lived in a freshwater lake, which contributes a well-dated fossil record to the ongoing research about the origin of freshwater species of Cymothoidae.

## Supplemental Information

10.7717/peerj.12317/supp-1 Supplemental Information 1 Information imported to R for morphometric analysis. A list of the species included and publications from which the additional illustrations were redrawn.

10.7717/peerj.12317/supp-2 Supplemental Information 2 The R code used for the morphometric analysis.

10.7717/peerj.12317/supp-3 Supplemental Information 3 The variation in the principle components (PC1–PC10) visualised in a boxplot.

10.7717/peerj.12317/supp-4 Supplemental Information 4 Visual representation of the mean shapes of each ontogenetic stage (immature, male and female) from all analysed specimens. Colour indication of the Euclidean distances of where the most variation to adult female representatives occur. Shades of red represent the highest degree of variation between immatures and adult female specimens, while orange and yellow represent variation to a lesser degree.

## Additional Information and Declarations

### Data Availability

The following information was supplied regarding data availability: The raw data is available in the Supplemental Files.

### New Species Registration

The following information was supplied regarding the registration of a newly described species: Publication LSID: urn:lsid:zoobank.org:pub:C38FC926-EEC4-45F8-8CBB-3639D845C4DA. *Parvucymoides* gen. nov. LSID: urn:lsid:zoobank.org:act:DE6F26BC-87E1-43B8-BDF9-47B25537627C. *Parvucymoides dvorakorum* sp. nov. LSID: urn:lsid:zoobank.org:act:485FBA58-F578-48A0-AD3C-D93991C6A8D3.
