# Supplementary figures and images for: Description and ontogeny of a 40-million-year-old parasitic isopodan crustacean: Parvucymoides dvorakorum gen. et sp. nov
Source: PeerJ. 2021 Dec 9;9:e12317. doi: 10.7717/peerj.12317 (PMC8667724; doi:10.7717/peerj.12317)

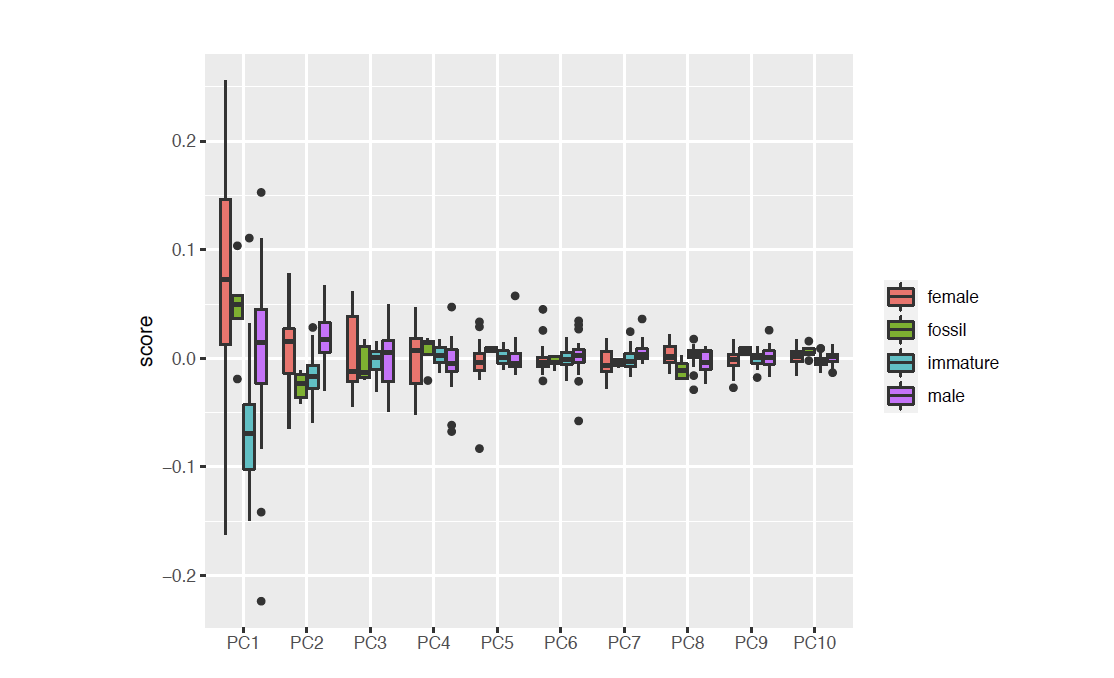

Supplement: Supplemental Information 3 [file peerj-09-12317-s003.png]
