# Supplementary material for: Description and ontogeny of a 40-million-year-old parasitic isopodan crustacean: Parvucymoides dvorakorum gen. et sp. nov
Source: PeerJ. 2021 Dec 9;9:e12317. doi: 10.7717/peerj.12317 (PMC8667724; doi:10.7717/peerj.12317)
Supplement: Supplemental Information 4 — Colour indication of the Euclidean distances of where the most variation to adult female representatives occur. Shades of red represent the highest degree of variation between immatures and adult female specimens, while orange and yellow represent variation to a lesser degree. [file peerj-09-12317-s004.pdf]

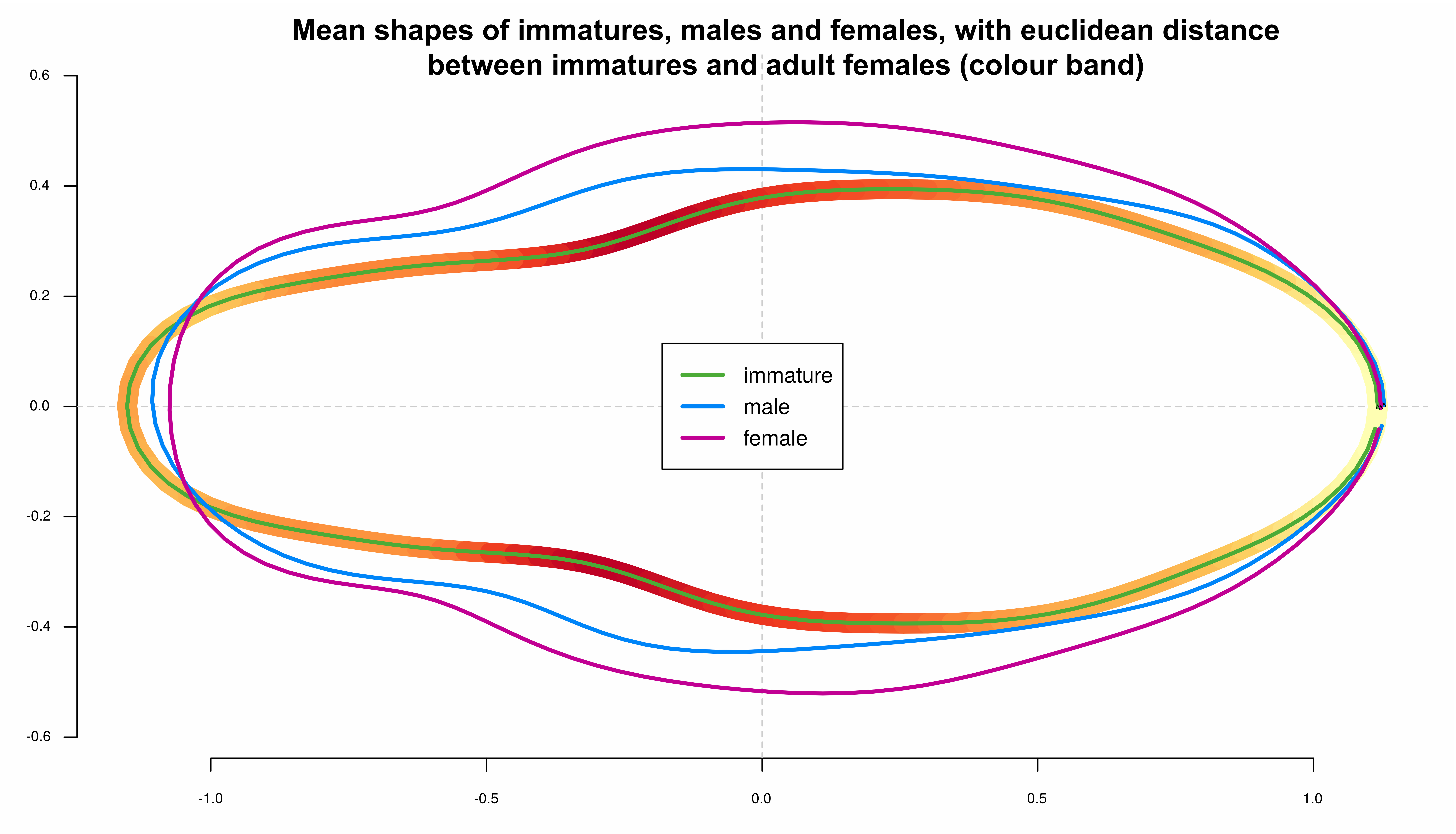
Mean shapes of immatures, males and females, with euclidean distance
between immatures and adult females (colour band)
0.6
0.4
0.2
0.0
-0.2
-0.4
-0.6
immature
male
female
-1.0
-0.5
0.0
0.5
1.0
